# Living on the edge: How to prepare for it?

**DOI:** 10.3389/fnrgo.2022.1007774

**Published:** 2022-12-14

**Authors:** Martine Van Puyvelde, Daisy Gijbels, Thomas Van Caelenberg, Nathan Smith, Loredana Bessone, Susan Buckle-Charlesworth, Nathalie Pattyn

**Affiliations:** ^1^Vital Signs and PERformance Monitoring (VIPER) Research Unit, Life Sciences (LIFE) Department, Royal Military Academy, Brussels, Belgium; ^2^Brain, Body and Cognition (BBC), Department of Psychology, Faculty of Psychology and Educational Sciences, Vrije Universiteit Brussel, Brussels, Belgium; ^3^Clinical and Lifespan Psychology, Department of Psychology, Faculty of Psychology and Educational Sciences, Vrije Universiteit Brussel, Brussels, Belgium; ^4^School of Natural Sciences and Psychology, Faculty of Science, Liverpool John Moores University, Liverpool, United Kingdom; ^5^Human Behavior and Performance Training, European Astronaut Centre, Cologne, Germany; ^6^Protective Security and Resilience Centre, Coventry University, Coventry, United Kingdom; ^7^Oxford Human Performance, Oxfordshire, United Kingdom; ^8^Human Physiology and Human Performance Lab (MFYS-BLITS), Human Physiology Department, Vrije Universiteit Brussel, Brussels, Belgium

**Keywords:** isolated, confined, extreme environment, ICE-environment, isolation, space-analog, Antarctica, training

## Abstract

**Introduction:**

Isolated, confined, and extreme (ICE) environments such as found at Antarctic, Arctic, and other remote research stations are considered space-analogs to study the long duration isolation aspects of operational space mission conditions.

**Methods:**

We interviewed 24 sojourners that participated in different short/long duration missions in an Antarctic (Concordia, Halley VI, Rothera, Neumayer II) or non-Antarctic (e.g., MDRS, HI-SEAS) station or in polar treks, offering a unique insight based on first-hand information on the nature of demands by ICE-personnel at multiple levels of functioning. We conducted a qualitative thematic analysis to explore how sojourners were trained, prepared, how they experienced the ICE-impact in function of varieties in environment, provided trainings, station-culture, and type of mission.

**Results:**

The ICE-environment shapes the impact of organizational, interpersonal, and individual working- and living systems, thus influencing the ICE-sojourners' functioning. Moreover, more specific training for operating in these settings would be beneficial. The identified pillars such as sensory deprivation, sleep, fatigue, group dynamics, displacement of negative emotions, gender-issues along with coping strategies such as positivity, salutogenic effects, job dedication and collectivistic thinking confirm previous literature. However, in this work, we applied a systemic perspective, assembling the multiple levels of functioning in ICE-environments.

**Discussion:**

A systemic approach could serve as a guide to develop future preparatory ICE-training programs, including all the involved parties of the crew system (e.g., family, on-ground crew) with attention for the impact of organization- and station-related subcultures and the risk of unawareness about the impact of poor sleep, fatigue, and isolation on operational safety that may occur on location.

## Introduction

*“Something is… different.”*

*“Good or bad?”*

*“Anything different is good.”*

*(from the movie “Groundhog day” Albert and Ramis, 1993)*

The duration of a round-trip to Mars is currently estimated to last up to 18 months. A few important factors in life sciences limit the possibility to expose humans to such long-duration flights, e.g., the effects of long-duration exposure to cosmic radiations, microgravity, and the “human factor”. To meet this human factor, the operator's resilience and adaptivity to isolated, confined, and extreme (ICE) environments, has been the focus of space agencies' sponsored research for a couple of decades with the aim of safeguarding operational safety during long-duration missions.

Although there is no unequivocal evidence showing a decrement in performance during space flight (Strangman et al., 2014), subjective reports of astronauts as well as real-life evidence on errors and omissions suggest that current experimental reports might be underestimating potential decrements (Manzey et al., 1995; Nechaev et al., 1998). Nevertheless, although it has been clearly shown that microgravity affects perception and psycho-motor coordination (Van Ombergen et al., 2017, 2021), the question remains whether attention and processing are affected as well. Indeed, whereas it is easy to conceive how microgravity disturbs psycho-motor coordination, through the different range of inputs from the proprioceptive, vestibular, and visual systems, it is far less obvious why the suppression of gravity would affect operational functioning through attention, reasoning, or accurate judgment. Hence, besides microgravity, space operators might be affected by the cumulative effect of multiple stressors (Manzey and Lorenz, 1998; Fowler and Manzey, 2000; Smith, 2021) such as isolation, confinement, increased workload, fatigue and circadian desynchronisation. Therefore, to disentangle these multiple stressors of an ICE-environment, research has been organized in several locations on earth utilized as space-analog environments (e.g., different types of polar expeditions, caves submarine missions and space simulation environments in Antarctica and non-Antarctic regions) (e.g., Sandal et al., 2006; Palinkas and Suedfeld, 2008, 2021; Van Ombergen et al., 2017; Golden et al., 2018; Mogilever et al., 2018). In comparison to real spaceflight, these space-analog ICE-environments share similar characteristics from a psychological point of view. That is, the combination of being isolated from family and friends and being confined with a small group of people on a limited surface but also being exposed to changes in the somatosensory stimulation due to specific physical characteristics of the natural environment (Casler and Cook, 1999; Sandal et al., 2006; Palinkas and Suedfeld, 2008). Moreover, ICE-environments have the advantage to be less expensive and less technically complex than a space research station (Palinkas and Suedfeld, 2021). However, every ICE environment, be it in space or on earth, as well as each crew and the engaged external organizations, have their specific characteristics which may nevertheless create marked differentiations (Palinkas and Suedfeld, 2021).

To give some examples of this diversity, on Earth, Arctic and Antarctic regions are characterized by higher latitudes which coheres with increased seasonality due to marked changes in photoperiodicity (i.e., the lack of a normal day-night alternance over an epoch of 24 h) (e.g., Friborg et al., 2012; Pattyn et al., 2018; Zivi et al., 2020). The observed consequences in studies in different Antarctic stations and Arctic trek expeditions over the years included sleep loss, impaired cognition, negative affect and interpersonal tensions and conflicts, a cluster of symptoms that has been labeled as the “winter-over syndrome” based on Antarctic winter-over studies (Palinkas, 1992; Palinkas and Houseal, 2000; Palinkas and Suedfeld, 2008). Indeed, in Antarctica, a circadian phase delay in melatonin secretion (e.g., Kennaway and Van Dorp, 1991; Yoneyama et al., 1999; Pattyn et al., 2017), poor subjective sleep quality, an increased sleep fragmentation, as well as a decrease in slow wave sleep (Natani et al., 1970; Paterson, 1975; Bhattacharyya et al., 2008; Pattyn et al., 2017, 2018; Mairesse et al., 2019) have been regularly reported (see Pattyn et al., 2018; Zivi et al., 2020 for a review). Besides latitude, altitude plays an important role as well. For instance, in Concordia station, the average pressure altitude of 3,800 m causes a condition of chronic hypobaric hypoxia which has been shown to be a pervasive disturber of sleep, overruling the impact of seasonality (Tellez et al., 2014, 2016; Collet et al., 2015). A relationship between both latitude and/or altitude and the winter-over syndrome has been illustrated in several studies by Palinkas et al. (e.g., Palinkas, 1991; Palinkas et al., 1996; Palinkas and Houseal, 2000) showing that latitude and/or altitude were inversely associated with the outcomes on the winter-over syndrome. Changes in photoperiodicity are present in space as well, although in a different way than on earth. For instance, in the International Space Station (ISS), astronauts have to deal with short sunrises 16 times a day because the ISS orbits the earth every 90 min (Dijk and Czeisler, 1995). In the Mars simulation stations, latitude varies between stations (e.g., ~65° in Flatline Mars, ~35° in MDRS). Moreover, altitudes are not so extreme that they cause chronic hypobaric hypoxia. Besides latitude and altitude, the duration of isolation varies over different missions (e.g., ~16 days in Lunares, 4–12 months in HI-SEAS) and the crew size in each station or polar trek can differ from 2 persons to 24 persons (250 in the largest stations) in long duration missions. Moreover, due to the type of mission and/or the station environment, sojourners will be challenged by high physical demands (e.g., Arctic polar trek expeditions) or they may be, on the contrary, almost physically inactive if they do not integrate sport in their daily schedule (e.g., the most isolated Antarctic stations during winter) (Palinkas and Suedfeld, 2008, 2021; Pattyn et al., 2018).

The specific psychosocial impact of isolation and confinement is also variable over the different types of missions, and although it is sometimes discussed in the margin, it may actually be a variable of greater importance than is often considered (Kanas et al., 2001, 2009; Golden et al., 2018; Pattyn et al., 2018; Somaraju et al., 2022). The psychosocial impact factor has important ramifications for several components of future space flights (Kanas et al., 2001, 2009). The combination of isolation and confinement in the specific situation where people work and live together for a longer period may trigger exaggerated reactions to (sometimes minor) job-related or personal daily events or disputes (Jehn, 1995; Jehn and Chatman, 2000; Suedfeld and Weiss, 2000; Palinkas and Suedfeld, 2008; Driskell et al., 2018; Golden et al., 2018; Somaraju et al., 2022). Being separated from family and friends, while not being able to escape from the crew when feeling the urge to, may block the capacity to put things into perspective (Palinkas and Suedfeld, 2008, 2021; Driskell et al., 2018; Golden et al., 2018) and to separate normal professional discussions from personal interpersonal tensions (Jehn and Chatman, 2000). For instance, these types of cumulating dynamics resulted during the “105-day Simulation of the Flight of International Crew and Space Station-1999” (SFINCSS-99) in a physical fight among crewmembers and sexual harassment (Vanhove et al., 2015). Concerning the latter, a recent study on gender experiences in women that were involved in an Antarctic Australian mission reported striking statistics, namely that 36% of the interviewees had observed inappropriate or sexual remarks to colleagues and 63% received these types of remarks themselves. Nevertheless, none of these women specified their experiences in open response questions (Nash et al., 2019). All these types of tensions, which usually remain “under the radar” during missions, may become a dangerous hotbed of further conflicts, but also a source of individual distress. For instance, in a recent study (Somaraju et al., 2022), it was shown how current-day relationship conflicts predicted next-day individual strain and vice-versa. Moreover, high workload worsened this negative spiral whereas low workload decreased the association (Somaraju et al., 2022); hence showing how the different levels of functioning on station may interact with one another. Furthermore, initial efforts to install a positive group cohesion from the start of the mission appear to be crucial. Not only has a crew more motivation and energy in the first half of the mission to build group cohesion, negative relations established early in isolation appear to remain stable over time of a mission (Sandal et al., 1995). This can be confounded by observable dips in crews' morale and cohesion in the second half (Wood et al., 1999; Palinkas and Houseal, 2000; Kanas et al., 2001, 2009; Sandal, 2001; Palinkas and Suedfeld, 2008; Supolkina et al., 2021) and third quarter of a mission (Bechtel and Berning, 1991).

Hence, when considering the literature, one can state that the existing variability over ICE-environments may be interpreted in function of the prominence of each of its three respective factors (isolation, confinement or environment) in which isolation and confinement may refer rather to the psychosocial characteristics and extremeness to the physical characteristics of the ICE-environment. Recently, the interaction between the isolation-confinement (IC) factor and the extremeness (E) factor of ICE-environments was highlighted, stating that the impact of both factors may mutually reinforce one another when individual vulnerabilities are challenged (Van Puyvelde and Mairesse, 2022). Indeed, when looking at fundamental research, several studies have shown that sleep curtailment accounts for operational deteriorations which are part of the daily performance of ICE-personnel. For instance, vigilance decrements combined with poor attention and concentration (Van Dongen et al., 2004), visuomotor disturbances (Van Dongen et al., 2004), decreased reaction time (Choo et al., 2005), poor memorizing and memory consolidation (Nilsson et al., 2005; Stickgold, 2005), impaired decision making (Killgore et al., 2006; Venkatraman et al., 2007) may threaten daily routine safety procedures, scientific efficiency and specific technical operations as well as job satisfaction, a factor that has been indicated as a crucial motivator in long-term isolation since other social roles in normal daily life are limited or eliminated (Natani et al., 1973). On the other hand, day-to-day worries about internal and external problems such as within-crew conflicts (Somaraju et al., 2022), harassment (Vanhove et al., 2015; Nash et al., 2019), problems in family context or missing loved ones (Palinkas and Suedfeld, 2008; Kanas et al., 2009; Temp et al., 2020) may amplify the environmental induced problems (e.g., poor sleep, seasonal depression). Finally, an indispensable factor is consciousness. That is most individuals are seldom aware, neither of their increasing fatigue and performance impairment (Van Dongen et al., 2004; Mallis and DeRoshia, 2005; Yoo et al., 2007), nor of their isolation impact (Suedfeld and Weiss, 2000), possibly endangering their operational functioning on the station (e.g., Nilsson et al., 2005; Killgore et al., 2006; Orzeł-Gryglewska, 2010; Brown et al., 2013; Boivin and Boudreau, 2014).

The risk for developing behavioral and psychiatric problems that may result in human errors exist in space and isolated analog missions (McPhee and Charles, 2009). For instance, some studies reported one incident per every 2.86 person-years for shuttle missions (Billica, 2000), one incident per every 1.3 person-years in the MIR station (Marshburn, 2000) and 0.44–2.8 incidents per person-year in submarines (Thomas et al., 2000). In Antarctica, 12.5% of the personnel were reported to meet DSM-IV criteria for mental disorders after deployment (Palinkas et al., 2004). Human errors in operational settings, including ICE-environment, can be life-threatening and extremely expensive (e.g., reports in nuclear plant management and civil aviation, pp. 82 HUMEX report: Study on the survivability and Adaptation of Humans to Long-Duration Exploratory Missions, 2000) (Horneck et al., 2003; Manzey, 2004), hence, to safeguard optimal adaptation of operational personnel in simulation missions is of indisputable importance. Therefore, all Space Agencies have created programs—based on human factors approaches from the aviation world—to train future astronauts in recognizing these potential stressors and acquiring a new range of behavioral skills to cope with them. However, in space-analog environments, these types of trainings are rather underdeveloped if not non-existing, as if the space-analog environment is considered as the arena of training itself. Nevertheless, the crew of these simulation missions are exposed to similar environmental difficulties and hazards. One exception—although not comparable with the exhaustive astronaut preparatory trainings—could be found for a few years at Concordia station, where a “Human Behavior and Performance” (HBP) training was temporarily supported by the European Space Agency (ESA) team. The training was based on the “International Space Station Human Behavior & Performance Competency Model” (Bessone et al., 2008a,b) in which individual and interpersonal competencies (e.g., self-management, communication, cross-culturalism, teamwork, leadership, conflict management, decision making, problem solving) are put forward as required capacities to participate in long duration missions.

Based on the given background, in the current study, we aimed at exploring the experiences of 24 persons that participated in short or long duration missions in an Antarctic (Concordia, Halley, Rothera, Neumayer II) or non-Antarctic [Mars Desert Research Station (MDRS), Flatline Mars (FMars), Hawaii Space Exploration Analog and Simulation (HI-SEAS), Lunares, Lunar, Mars 6, 160, 500] space-analog station or a polar trek expedition. We opted for a qualitative approach with semi-structured interviews. We used a qualitative thematic analysis (Braun and Clarke, 2006) in order to obtain a detailed insight in how these participants were trained, prepared, how they experienced the impact of the ICE-environment and what differences were reported in function of the variety of ICE-environments as well as the variety of provided trainings, culture of the stations and type of mission. With this in-depth bottom-up analysis, based on first-handed detailed information, we aimed at taking a new direction, adding to the agencies' quest for understanding the nature of demands encountered by ICE-personnel at multiple levels of functioning.

## Method

### Participants

The study was approved by the Commission for Medical Ethics of UZ Brussels (B.U.N. 1432011939886). Twenty-four sojourners (10 women) were interviewed. They sojourned either in Antarctica (Concordia, Halley VI, Neumayer II, Rothera, long duration, i.e., overwintering, hence minimum 12 months); or at a Mars space-analog simulation (MDRS, FMARS, MARS160, MARS500, HI-SEAS, long or medium duration); or at a moon space-analog simulation station (Lunares, short duration) or executed an Arctic polar trek (short or medium duration). We considered long duration as minimum 12 months, medium between 1 and 12 months, and short as >1 month. Their mean age at the start of their mission was 30 years (*M* = 30.00; *SD* = 6.85), the estimated mean age of the crew of their mission was 31 years (*M* = 30.90; *SD* = 4.10). The interviews had a mean duration of 80.33 min (*M* = 80.33; *SD* = 17.62). All the participants signed an informed consent before the interview started (see Table 1 for an overview of the different types of participating space-analog stations with characteristics).

**Table 1:** Overview of the (Antarctic) (simulation) stations and their most important characteristics as well as the number of interviewees that sojourned there.

	**Location**	***n***	**Establishment**	**Duration mission**	**Latitude**	**Altitude**	**Landscape**	**Other characteristics**
Antarctica	Concordia	7 (2F)	Franco-Italian Constructed between 1999 and 2005	Winterover >12 months	75°	3,233 m (3,800 m)	No fauna No flora	Extreme remote Chronic hypoxia Mean temperature −52° (extreme: −80°)
	Neumayer III	1 (M)	German Neumayer I 1981–1992 Neumayer II 1992–2009 Neumayer III > 2009	Winterover >12 months	70°	Sea-level	Coastal Fauna (penguins, seals, birds)	Since Neumayer III aboveground Mean temperature −16° (winter: −24.9°)
	Rothera	1 (F)	British Phased construction since 1975	Winterover >12 months	67°	Sea-level	Flora: lichen Fauna: birds, penguins, seals	Mean temperature −3.7° (winter: −6.7°)
	Halley VI	1 (M)	British First established 1956, operational since 2012	Winterover >12 months	75°	Sea-level	No flora Occasional penguins	Mean temperature −20° (winter: −31°)
Non-Antarctica	MDRS - FMARS	4 (2F)	US Built first 2000 Utah	< 12 months	38°-75°	N/A	Martian landscape	Simulation station
	HI-SEAS V	5 (2F)	US – Hawaii Built 2011, first mission 2013	4–12 (meanly 8) months	20°	2,500 m	Martian and moon landscape	Simulation station
	MARS 500	1 (M)	Russian-China Experiments between 2007–2011	520 days	55°	N/A	Space simulator	Simulation station
	LunAres Research Station	2 (2F)	Poland near Piła Established in 2017	2 weeks	53°	N/A	Moon landscape	Simulation station
Arctic Polar trek	N/A	2 (1F)	N/A	1–5 months	>60°	N/A	Remote with variable fauna-flora (e.g., polar bears)	Trek mission

### Procedure and design

This study was part of a project supporting ESA's “Human Behavior and Performance Training” at the European Astronaut Centre in Cologne with the goal to provide guidelines for future participants in ICE-environments to mitigate psychological stressors and improve crew resilience in small teams. The participants were recruited using a combined purposive and snowball sampling approach. Participation was on a complete voluntary basis with interviewees made aware that they could withdraw at any point.

Each interview lasted between 60 and 90 min and was completed either in person, or through audio or video link, as preferred by the interviewee. The interview questions consisted of four main sections, i.e., 1/demographics and mission specifics, 2/training and personal preparation before departure, 3/coping strategies for challenges in small teams and, 4/open advice for future expeditioners. The interviews were constructed through an iterative explorative discussion between operational experts from the Human Behaviour and Performance team of the European Astronaut Centre in Cologne, working with analog, space and ground crews, and academic experts in adaptation to extreme environments.

### Materials

The interviews were transcribed, coded, and analyzed using NVivo (^©^QSR International, release 1.6). Quantitative analyses were performed using SPSS (version 27).

### Qualitative analysis

**Figure 1:**
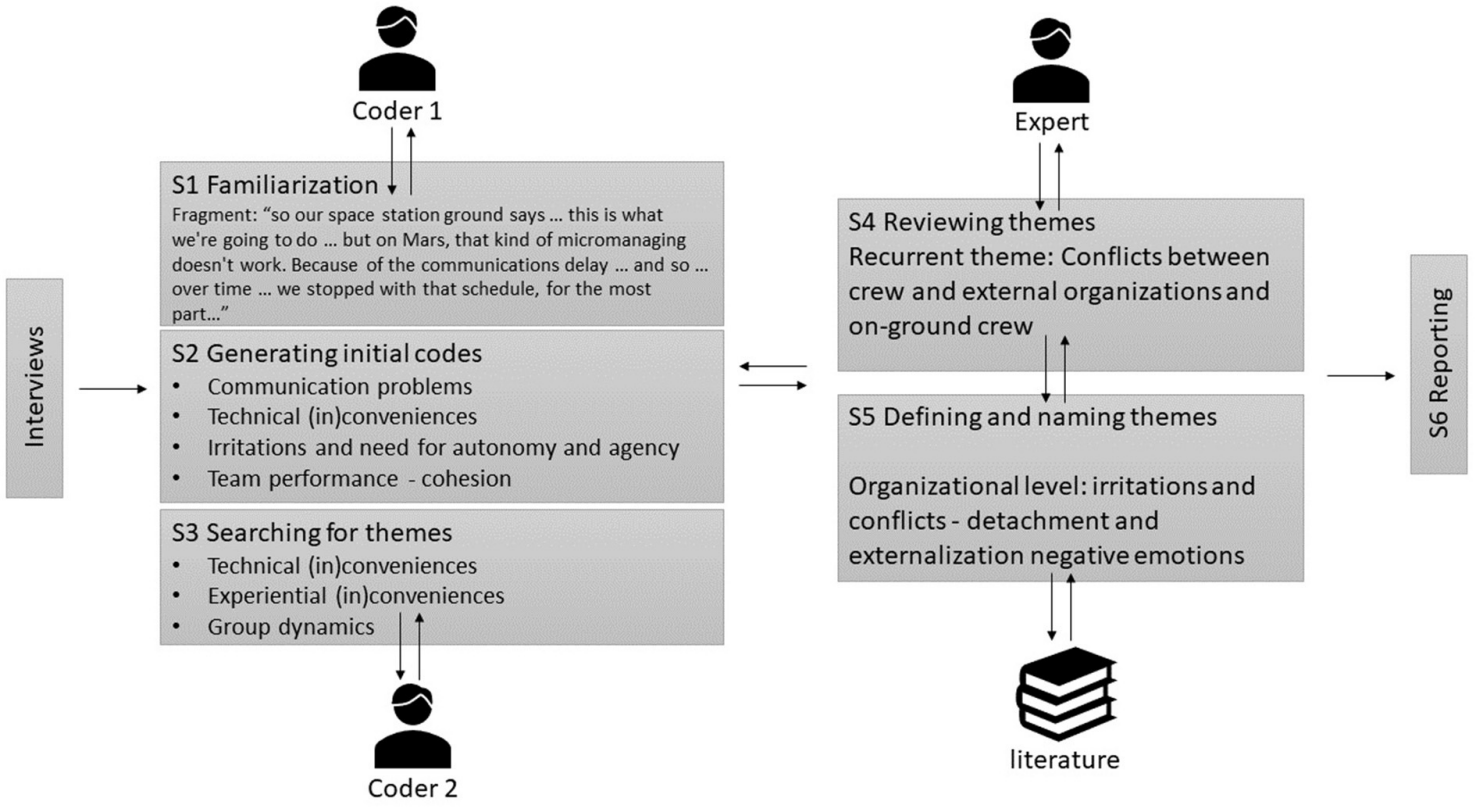
Illustration of the applied methodology of Braun and Clarke (2006) with a triangulation method (Carter et al., 2014). The content is labeled in initial bottom-up driven codes, independent from theoretical concepts or models by two coders. Then these codes are discussed to create more generalizing themes. Then the themes are cross-checked with the literature and an expert, having extensive experience with the topic to generalize a final coding scheme before reporting data.

We used the inductive thematic analysis approach of Braun and Clarke (2006) expanded with a triangulation method (Carter et al., 2014) to ensure truth value (see Figure 1). This non-linear recursive method is based on six phases of analysis, which need to be flexibly applied and that demand a continuous back and forth movement through data. It is an inductive bottom-up approach that takes the data as a starting point. In accordance with Braun and Clarke (2006), the first phase was a familiarization with the data by transcription, reading, listening, and note-taking. In the second phase, we generated 406 initial codes in a bottom-up manner by, on the one hand, systematically coding salient features of the data and, on the other hand, collecting data relevant to each code. Subsequently, in phase three, we identified the potential overarching themes to organize the data into. For this, we based interpretations on human factors and group dynamic models. In the fourth phase, the themes were reviewed by checking whether the themes cohered with the coded parts as well as the entire data set. Then, in the fifth phase, the codes were defined, specified, and labeled to prepare the final sixth phase or results section. The analysis process in summarized in Figure 1.

To ensure truth value (Krefting, 1991) or credibility (Lincoln and Guba, 1985) we applied the triangulation method (Carter et al., 2014). Truth value or credibility refers to how confident the researcher is with the truth of the findings based on the research design, informants, and content, from which the data were drawn or persons who are familiar with the phenomenon being studied). By applying the triangulation method, we aimed at increasing the validity of the results by a crosscheck over different sources, i.e., methods, literature, concepts, and researchers. Triangulation of researchers refers to the simultaneous participation at the analysis process of at least two researchers (Carter et al., 2014). Hence, we completed the first bottom-up stage by two researchers whereas in stage three we added a third researcher with extensive experience in both short and long duration missions in Antarctica to review and reflect on the resulting themes, categories, and interpretations. According to Sandelowski (1986), a qualitative study is credible when it presents such accurate descriptions or interpretations of human experiences that individuals who shared similar experiences would immediately recognize them.

## Results

### Truth value

The third researcher recognized and confirmed the validity of the identified themes through analysis as well as the final interrelatedness between the different components. Moreover, the themes appeared to correspond with or expand previous literature.

### Human behavior and performance model

**Figure 2:**
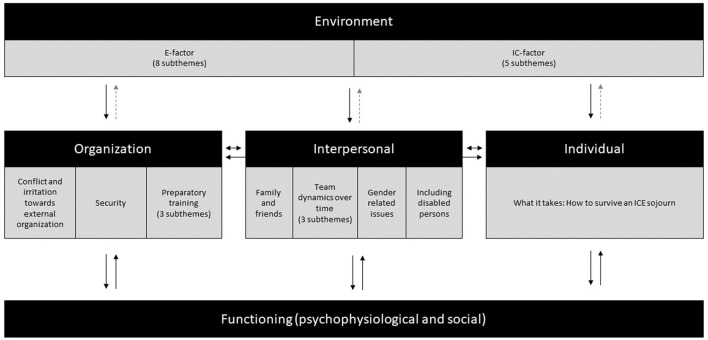
Human behavior and performance model, illustrating how the ICE-environment shapes the impact of organizational, interpersonal, and individual systems, thus influencing the function of sojourners. We interpreted the impact of the environment with its IC- and E-factor as (quasi) unidirectional, hence the dotted line to indicate that potentially, under exceptional circumstances, the other factors might back influence the environment.

Figure 2 displays the Human Behavior and Performance Model that illustrates how the ICE-environment (environmental level) shapes the impact of organizational, interpersonal, and individual systems, thus influencing the functioning of sojourners. We observed an impact of the environmental level in terms of the extremeness (E-factor) on the one hand and psychosocial aspects (IC-factor) on the other hand and interpreted this environmental impact as (quasi) unidirectional, hence the dotted line to indicate that potentially, under exceptional circumstances, the other factors might influence the environment back. Each level (environmental, organizational, interpersonal, and individual) will be discussed in the result section below.

### Environmental level

All the interviewees discussed the environmental level and its impact. The E-factor (extremeness) describes the physical characteristics of the ICE-environment and its consequences. The IC-factor (isolation and confinement) describes the psychosocial consequences imposed by isolation and confinement.

#### E-factor

##### The landscape: Monotony and harshness

In the long Antarctic missions, the monotony, the harshness of the landscape without colors and close to no fauna or flora is one of the most recurrent features said to impact the interviewees' behaviors and habits: “*it's really like being in one white sheet of paper”*. In Arctic polar treks, an alternation between monotony and high or dangerous excitement was described, for instance, when a polar bear crossed their path. In the stations, quite some interviewees described how they attempted to be creative to combat monotony, whereas a few others claimed that every day was different without doing any effort (e.g., the cook makes a difference with their daily menu). In the non-Antarctic missions, although the high fidelity of a Martian landscape was praised, the interviewees missed colors (everything was red or white when it snowed) and monotony was often discussed. In polar treks, when monotony was combined with hard physical endurance, the expeditioners attempted to overcome these days by imaginary games, sticking to important dates, and being proud of what had been reached so far (see also Supplementary Table 1).

##### Changes in sensory stimulation and the sensory system

Isolation, monotony, and/or changes in photoperiodicity in high latitude regions were reported to induce oversensitivity in the sensory system and/or a craving for certain types of stimulation and variation. Fourteen out of 24 interviewees testified about changes in the visual system. The extremeness of summer (constant light) and winter (constant darkness) or being entirely cut from sunlight (Lunares) was reported to impact the eyes' sensitivity and mood. Although some interviewees indicated that winter was the core of the expedition and thus should be enjoyed, the sensory deprivation created a craving for colors (certainly green) and odors. Some other participants testified that they became more sensitive to noise (Lunares). To satisfy this craving, pictures with nature were used and sometimes scented oils. However, the latter appeared not effective because the sense remained artificial, whereas the body is rather missing the natural smells of flowers and woods (see also Supplementary Table 1 and Section Gender-related Issues).

##### Hypoxia

The Antarctic station Concordia is known for its conditions of chronic hypobaric hypoxia, which impacted most of the crew members in cognitive functioning and sleep (see also Section Sleep and fatigue).

##### Physical activity

In the non-Antarctic long duration missions, the sojourners reported to install structural physical activities, analog to space missions (e.g., 1 h/day), to avoid general health issues and to stimulate endorphin secretion. In the Antarctic stations, some sojourners executed some sport or yoga whereas others did not. The polar trekkers executed high levels of physical exercise (see also Supplementary Table 1).

##### Sleep and fatigue

The greatest sleep problems were reported in the Antarctic station Concordia. The participants appeared to be not well informed beforehand about the impact of hypoxia and the lack of photoperiodicity on sleep and fatigue because they all indicated that they thought it might have been something to do with pressure or melatonin but not one interviewee stated this by knowledge: “*I think that mainly it was because the oxygen … Just what the reason may be? And I'm not sure of course, maybe”*. In the bad sleepers, sleep deprivation induced chronic fatigue of which some of the participants became slightly aware during their mission. In HI-SEAS, some persons slept well, with a mean of 8 h per night, in MDRS, FMARS and Lunares sleep was not obvious, and, in the Arctic polar treks, sleep loss is accepted as an occasional and inevitable part of the expedition. For those interviewees, the biggest enemy was cold, which however, could also interfere with sleep. To combat fatigue, the polar trek expeditioners mentioned to rely on willpower and the consciousness they must take care for one another. In the non-Antarctic simulation stations, sleep problems were mainly ascribed to a lack of sound isolation in the walls between rooms. Suggestions to combat poor sleep were very mixed. Some interviewees swore by maintaining a strict day-night schedule whereas others advised the opposite, going to sleep whenever they felt the need. Some persons tried using light therapy and melatonin with mixed results (see also Supplementary Table 1).

##### Design and decoration

The design of the building is a direct consequence of specific environmental factors and technical necessities and has an impact on the daily experience of life in the station (e.g., station with almost no windows or non-pragmatic stair design). Decoration was an often-discussed topic, i.e., the importance of personal decoration that reminds of home as well as group decoration that creates bonding between team members. Furthermore, a lack of privacy and silence in some stations was regularly mentioned (see also Supplementary Table 1).

##### Technical restrictions: Asynchronous communication and internet issues

A temporal mismatch in communication between sojourners and both the professional and personal external world had an enormous impact on the daily life, in such a way that some of the interviewees desired to be better prepared for that. They claimed not to have realized how hard this would be for them and their family, and communication problems were reported as a risk for mood and resilience (see also Supplementary Table 1).

##### Potential life-threats due to technical issues

In the station missions, some communication issues or technical issues were told to lead to situations in which the sojourners started to feel insecure, pondering for instance how the lack of a solution could become life-threatening in the long term (e.g., temporary absence of water supply, defect in heating system with −70° degrees outside). The knowledge that external on-site assistance is impossible was reported to create anxiety in some interviewees (see also Supplementary Table 1).

#### IC-factor

##### Loneliness, home sickness, and contact with the external world

The degree in which interviewees missed the outside world, which persons they missed and how much and how they coped was very different for everyone. Some persons anticipated this demand by putting a lot of pictures in their room whereas others just tried to avoid this because it felt like torture to see the pictures of the persons they were missing. The frequency of phone calls with loved ones varied greatly over sojourners, ranging between daily and twice a year. Some interviewees explained the confusion about feeling very lonely when surrounded by and living together with a group of people. One interviewee explained that she instigated the use of (unsexual) touch such as massage or a hug during the mission to cope with difficult moments and how that worked fine without creating sexual tension (see also Supplementary Table 2).

##### Isolation and the goldfish bowl effect

All the interviewees described situations in which isolation and confinement made them lose their capacity to put things into perspective causing small events or conflicts to become disproportionate: “*You get angry very quickly you might become very happy quickly, much more than in regular life*”. Although most of them were informed about the impact of isolation, they did not always feel prepared for it. The interaction between the isolation aspect and interpersonal conflicts was a dangerous combination that could disturb the individual functioning as well (see also Section Interpersonal level). When persons got stuck in their restricted “goldfish bowl” perspective, they could not see any way out of the current situation. This led to dangerous situations. For instance, as described by an interviewee, a person that wanted to escape the station to isolate himself, went outside in the harsh and dangerous cold landscape and was found in the morning with serious hypothermia. It was never clear whether the person wanted to commit suicide. Another example is that some persons did not realize that being literally physically isolated from external help—without medical evacuation possibilities, if necessary—comprised a real risk (see also Supplementary Table 2).

##### Salutogenic effects

Antarctic missions, simulation missions and expeditions, they are all a unique, and often, a once in a lifetime experience. Feelings of gratitude to be part of that experience were expressed by 16 out 24 interviewees and were clearly a beneficious counterpart for moments of difficulty during the mission:

“*How dare you be devastated, you've had 21 days on the ice, you've had three polar bear encounters, you've, you've had the most amazing experience, I'm not from a rich background, I don't have any money. And there are, you know, to be able to do that is a privilege…*.”

##### The importance of food

To break out of monotony, and to avoid that every day looks the same, food and the menu was a major event in every station. Moreover, during winter there is no supply of fresh fruit and vegetables anymore. The station crews and cooks often organized thematic food evenings, cooked a person's favorite meal etc. Food was more than about eating, it was a time for discussion, open conversations, a time to meet, and in some crews, it was crucial that everybody was always part of the mealtime (see also Supplementary Table 2).

##### Spending time

Time was experienced in a particular manner. Firstly, the sense of time over the course of the mission was salient. In long duration missions, the second part of the mission was often experienced as more difficult, however, the third quarter effect was not confirmed. Secondly, it was reported that distributing time between work, free time alone and free time in group was a difficult balance to keep. Thirdly, in the team, the group dynamics followed a process of several stages over time (see Section Interpersonal level) (see also Supplementary Table 2).

### Organizational level

Aspects situated at the level of the external organization were explicitly discussed in 23 out of 24 interviewees. As will be clear below, they are intertwined with and have an impact on the interpersonal and individual levels. Organizational factors clearly impacted both small and big daily events of the crew on stations, causing the unpleasant feeling that they lost control whereas in polar treks, the sponsoring organizations beyond or the loyalty to the scientist for which they collected data could be, at the contrary, an extra motivational factor to continue when time was hard:

“…*how I get through all the time you have an honor to be here, you've left people behind your family who are trusting on you. There are people that have sponsored you, there are people out there that you've got to service, there are people relying on you. And it's okay to fail. But you can't say I gave in, you took this job, nobody asked you to do it, you took it. So, you have a duty*.”

#### Conflicts and irritations vs. loyalty toward the external organization

The experiences from the station sojourners stood in stark contrast with those of the polar trekkers described above. In the interviews of the station sojourners, communication with the external organizations or on-ground crews sometimes was often experienced as stressful. The most described reasons were the asynchronous (temporal mismatch) communication which complicated the exchanges, cultural differences, an ambiguity about who was responsible for what, and the idea that some external organization members had no or too limited experience with the living conditions in ICE-environments. From the station sojourners' perspective, responses to urgent issues either came too late or were inappropriate which could lead to endless discussions or explanations. For instance, some interviewees described that the on-ground crew comprised volunteers, working in 4 h shift blocks. Hence, once an issue started to be solved, it was the next shift group's turn, meaning that the communication needed to be re-opened again, sometimes recommencing from the very beginning.

Some interviewees reported that there was an over-attention before departure in terms of mental and physical health screening whereas there was a lack or no screening during the sojourn or afterwards to ensure beneficial return and re-integration post-mission: “*I think they basically, they look at you as a worker. And they are not more focused on the… a lot of… on people*”. This feeling was amplified by the fact that they never received feedback about the experiments they have been participating in during their sojourn (see also Supplementary Table 3).

In the non-Antarctic simulation missions, it was regularly claimed that the work planning provided by the external organizations lacked a reality check in terms of the station living conditions (e.g., sleep deprivation) and the workload of the crew on board: “*And we are telling them, we can't do more, because we're all like, lacking sleep, and it's very bad… And they are like, oh, but we have this and that…It was just very hard*”. Moreover, for some interviewees, it was not always clear why a simulation context was needed for certain experiments selected for implementation which created frustration on peak moments when the team started to feel at the end of their strengths (see also Supplementary Table 3).

#### Security

Basic medical security was not always prepared or in place on arrival which created sometimes small medical issues and could induced feelings of insecurity:

“*Sometimes I get to hear about simulations or even see simulations that are starting from scratch and they're making the same mistakes that have been made so many times instead of learning from the things that have already been found*.”

#### Preparatory training

##### Training

Training to prepare personnel for the isolated missions was highly variable. Some sojourners received a job-specific (e.g., rescue training for medical doctor) and/or job non-specific technical (e.g., glacier training, first aid, firefighting) and/or job non-specific non-technical trainings (e.g., communication training, teambuilding). “*Preparing the team is the success of the team*”, one interviewee said, adding, “*I'm not sure that this … like the people from the organization are already thinking about this seriously*”. The opinions concerning the trainings in general were mixed. Only two persons (one medium and one long simulation mission) fully agreed that they had enough trainings and that these trainings also prepared them well for their mission. The largest number of the interviewees indicated that they would have liked to have been better informed in advance. Some participants received a booklet which they appreciated but should, according to them, be expanded and elaborated for future expeditioners. Finally, during training, more attention should be paid to gender-differences (see Section Gender-related issues).

In the non-Antarctic simulation missions, a considerable number of the interviewees indicated that they did not receive training, or their training was part of the selection procedure that took place 2 years before departure: “…*there was no formal training … No, I was honestly not happy with the way they prepared us*…”. For those that received training, the trainings were in general well received regarding content but were found sometimes too short or too hectic in the organization. Participants appreciated the extended communication training but, it was mentioned that an essential part was lacking, namely the training of the on-ground crew. Although the on-ground crew might have received a training of their own, they were not trained together with the expedition team and thus not prepared on the specific dynamics with their team in particular, whereas these were exactly the persons they needed to communicate with:

“*But … it didn't include all the, the remote team that was, that, that's also a big part of the mission, you know, because we mostly communicate with them when we have a particular issue, technical issue or stuff like that … and these people were not prepared to, to make this mission happen*.”

The difficulty to provide a well-tailored training program was a topic of discussion because it is difficult to fully understand the impact of isolation and confinement before having experienced it yourself: “*I was warned, but maybe not warned enough*”, as it was put by one interviewee. Hence, on the one hand participants had the feeling they lacked training and on the other hand some of them wondered whether one could be really prepared. Some trainings were particularly appreciated in terms of team-bonding. Most of the interviewees, however, desired more elaboration in a concrete, explicit and practical manner (e.g., simulation, exercises, practical advice). However, also here, one interviewee wondered whether the tensions and problems that occur during mission can be formalized and concretized into a preparatory training or advice: “*Because if you're aware of what's going on, you're you can be, you know, prepared for that*”.

Finally, one interviewee of a short simulation mission mentioned that trainings were not mandatory, whereas they should have been. If not, this may stimulate that some persons get to know one another already better than others on beforehand, risking clique-forming (see also Supplementary Table 3).

##### Inter-crew exchanges and packing

All the interviewees agreed it is important to discuss with former crews and most of them wished they would have received more concrete information on what to pack, how to prepare and how to organize. Some interviewees indicated that what to pack may differ by age group, profession, role and gender, information that could be prepared by the external organization. Further, 23 out of 24 interviewees emphasized to bring something personal that reminds home (e.g., a scent, pictures, gifts, music) (see also Supplementary Table 3).

##### Preparation for family and friends

Some interviewees reported that information sessions for the family would have been useful. For instance, topics such as the frequency of contact, what information do you share, the emotional difference between a phone call and a mail and (dis)advantages of each, how to handle bad news from the external world (e.g., death of a family member): “*And none of us had previous experience there. So, we didn't know anything of what we should expect during FMARS*” (see also Supplementary Table 3).

### Interpersonal level

#### Family and friends

See Sections Technical restrictions: Asynchronous communication and internet issues and Preparation for family and friends.

#### Team dynamics over time

Teams went through different subsequent stages, starting by getting to know one another during teambuilding trainings in the preparatory stage over periods of conflicts that needed conflict resolution toward the final reorganization and ending of the team.

##### Getting to know during crew selection and team building

Crew selection and teambuilding was the place and moment where the team members met one another for the first time. Some crew members of non-Antarctic long simulation missions did exercises and games to stimulate exchanges during crew selection which was well received (e.g., discussion topics such as “how would you respond in this particular situation?”, “what is important in life”). The first meetings were also an opportunity to get insight in the interests of the others, which was important to connect later during mission (e.g., you could read about certain passions of the others) (see also Supplementary Table 4).

##### From conflicts and frustrations toward conflict resolution and teamwork

Every team went through certain conflicts (from small to big conflicts that impacted the entire group). Some interviewees indicated how the role of the station leader is crucial in these cases to protect the group, i.e., a station leader hesitative in taking authority may destroy a group dynamic very quickly. Conflicts could be driven by culture differences, personal disagreements or professional disagreements and it was often reported that isolation was an amplifying factor in this. Moreover, conflicts could be internal (i.e., between crew members) or, as discussed above, external (i.e., toward the organization or on-ground crew).

To overcome these conflicts, team members search for rules and strategies that suited the group. The main goals of these rules were to avoid those conflicts becoming too big and to prevent them in the future. An often-recurring strategy was to take meals together with the entire team as a structured moment for discussion and exchange. The HI-SEAS preparatory training was praised for conflict training.

The best reward for conflict solution was to benefit from real and genuine teamwork. All interviewees described the efforts that they put into reaching a stage of at least being able to rely on one another and to strive for certain moments of cohesion. For some teams, cohesion was strong, and teamwork was passioned while caring for one another. Within Arctic polar trek teams, reliance and trust were considered even more important and indispensable (see also Supplementary Table 4).

##### End of the mission

Ending the mission was a stage full of mixed emotions and often a quite hectic period. On the one hand, people desired departing but on the other hand, saying goodbye after such a long and intense period was said to be difficult and emotional as well. Moreover, this period also announced a new period of uncertainties about re-integration and one's post-mission professional future. On top of these uncertainties, the time to return home was not always obvious. For instance, the (boat) trip back could be unpredictable and the date of return was dependent on weather conditions (which was deemed acceptable by the interviewees) but also other organizational issues (which was deemed less acceptable). Persons often preferred returning by ship over plane to have a period of transfer and readaptation, to avoid entering in the crowdy world from 1 day to another.

The final period was also heavy in terms of work, e.g., finalizing the scientific work, cleaning, and preparing the station for the subsequent crew. However, the most difficult was removing personal items to give the floor to the next crew and to definitively close this period. Four teams out of the 24 involved in the current study are still in regular contact by WhatsApp and email. One team meets annually. One person started a relationship with another crew member after the mission, there is no information about the couples during mission and three close female-female friendships were mentioned.

#### Gender-related issues

Two teams were male-only crews, all the other were mixed-gender teams. The small mixed-gender teams were rather balanced (e.g., 2 women vs. 3 or 4 men), the larger Antarctica teams were not (e.g., 2 or 3 women vs. 13–23 mean). According to all the women, differences in gender should be handled from the preparatory stage onwards, in logistic aspects such as packing as well as group dynamic aspects such as how to position oneself within a team.

Flirtations and romantic relationships occurred once in the small mixed-gender teams and often in the large ones. In some cases, they caused problems, when the relation broke up and feelings of jealousy entered the group or as mentioned by one male, when a woman would start flirting with everybody. In other cases, interviewees testified that a relationship could install stability in the team (e.g., a couple may support one another and invest in the team at the same time, creating group cohesion). The seriousness of commitment of the romantic relationship seemed to define how well it was received by the group.

Verbal harassment or unwanted flirtations from men to women were mentioned—mostly briefly—in three out of 10 women. One woman specified that, at an official level, people are not allowed to talk about it:

*Yeah, this sort of sexual politics on base were a real thing. And they were a pretty major thing. And yet, on an official level, you were never allowed to talk about it, you know …It's just that, you know, you, the management preferred to assume that that was never an issue* (see also Supplementary Table 4).

#### Inclusion of disabled persons

In one long MDRS mission, it was decided to take on board a disabled person to train the functioning of the team when having to take care for a disabled person. The idea beyond was that during a mission an accident can quickly occur and when someone gets injured seriously, the mission needs to continue. The respective person was blind and had lost both hands except the thumb and index on his right hand. The crew found it extremely interesting to adapt in both a professional and personal manner to include the disabled person in such a way that he felt fully accepted and could be highly efficient for the team (see also Supplementary Table 4).

### Individual level

The environmental, organizational, and interpersonal factors interacted with personality traits and features they developed through the mission at the individual level of each crew member. The interviewees shared what, according to them, were important characteristics to possess or to develop to function well in an ICE-environment.

#### What it takes: How to survive an ICE-sojourn

To survive an ICE-sojourn as an individual within a group, 23 out of 24 interviewees emphasized the importance of an open and transparent but well-timed communication in which one must find a balance between not expressing immediately what irritates and not waiting too long neither. Participants, quite often put the wellbeing of the group on the first place, before their own wishes or expectations (a collectivistic approach). Moreover, a lot of interviewees described how they learned to reflect and manage their own function in the group, being aware of when to put aside their own opinion or desire. Half of the interviewees underlined how one cannot be aware enough of the impact of isolation (i.e., the formerly mentioned goldfish bowl effect) and that each crew member should repeat daily, as a mantra, the range of dangers that isolation can cause, in order to avoid tumbling into it. A recurrent advice was to install mutual helpfulness in the group and to remain interested in the other person. To be able to accept the other, it is important to be able to accept yourself as well, was said by some interviewees. Moreover, the importance to remain down to earth was underlined and how a focus on the job and one's professional ethics can help in that. The female participants indicated that gender issues in ICE-environments should be addressed. Nine interviewees emphasized the importance of positive psychology, how positive emotions as well as humor are contagious and can contaminate the other members of a group in an upwards dynamic. And finally, the polar trekkers underlined that one may never forget the chance they have to partake in this unique experience in an exceptional environment (see Supplementary Table 5).

## Discussion

In the current study, we conducted 24 semi-structured interviews with persons that sojourned in a space-analog ICE-environment (i.e., Antarctica, MDRS, FMARS/MARS500, MARS160, Lunares, polar trek) and analyzed them using a thematic bottom-up analysis (Braun and Clarke, 2006).

The results showed that for all the interviewees, there was an unignorable impact of the ICE-environment. The interviewees described how the ICE-environment impacted their organizational, interpersonal, and individual functioning, imposing a psychophysiological blueprint that affected their behavior and performance during the mission. Firstly, sleep difficulties were recurrently reported with the severest complaints in the Antarctic station Concordia. It is well known that sleep problems remain one of the main issues in Antarctic sojourners due to the interaction between higher latitude and marked seasonality (Pattyn et al., 2018). Moreover, research has shown that, in Concordia, sleep problems are indeed even worse due to the high-altitude conditions (average pressure altitude of 3,800 m) that induce chronic hypobaric hypoxia (Tellez et al., 2014, 2016; Collet et al., 2015). In Neumayer II, in the period of the interviewee's mission, the station was still built underground hindering summer light to reach the inside of the station (Steinach et al., 2016). In the Mars simulation stations, sleep problems were reported as well, but they were rather ascribed to sound isolation problems between rooms in the building. The interviewees of the polar trek expeditions did not complain about sleep, considering this as an occasional discomfort that was part of the job. Moreover, according to them, the extreme physical activity during the day compared with the sedentary life in certain stations may help against sleep loss. Nevertheless, increasing physical exercise in Concordia would not be a solution since previous research showed that exercise exacerbated the respiratory sleeping problems (Tellez et al., 2016). Poor sleep is known to threaten operational safety since it affects the individual's cognitive flexibility (Alhola and Polo-Kantola, 2007; Palmer and Alfano, 2017). Moreover, several studies have shown that its impact is rarely identified by the affected individual itself (Van Dongen et al., 2004; Yoo et al., 2007; Howell et al., 2010; Garland et al., 2013) due to decreased cortical-subcortical brain connectivity (Yoo et al., 2007). In the current study, the greatest part of the interviewees reported that poor sleep did not intervene with their performance, which may thus be part of their symptomatology (Van Dongen et al., 2004; Yoo et al., 2007), once again illustrating the potential insidiousness of the phenomenon. For instance, in terms of operationality, even one night of sleep loss has shown to disrupt the ability to incorporate new information, to reflect on different task approaches, and to learn from prior mistakes (Harrison and Horne, 1999). Moreover, in an ICE-context, this danger may even be amplified, when a lack of awareness of fatigue is combined with the impact of isolation and confinement.

Indeed, the psychological consequences of the monotony, the isolation, and the confinement were the most cited environmental impact factors throughout the interviews. These three factors appeared to be closely connected and may be interpreted as the result of an umbrella of sensory deprivation, impacting both the individual and interpersonal psychophysiological functioning of the sojourners. As regularly reported in the first studies on Antarctica (e.g., Cook, 1900; Strange and Youngman, 1971; Natani et al., 1973; Blair, 1991; Rothblum et al., 1998) and as testified again in the current interviews, the monotonous landscape, the constant cold and wind, the daily white-outs, combined with engaging with the same crew members every day and the continuously nagging feeling of missing family and friends can make every day look the same (Ender, 2012). As a result, most of the interviewees reported to experience a crave for variation in color, odor, and activity and an increased sensory sensitivity. This increased sensory sensitivity ties in with physiological results, as shown in a study by Kawasaki et al. (2018) who found increased retinal sensitivity to blue light after long-term daylight deprivation in the Antarctic stations Concordia and Halley VI.

Boredom and monotony have often been indicated as one of the highest sources of risk for long duration missions (e.g., Rothblum et al., 1998; Kanas et al., 2001; De La Torre et al., 2012; Gunderson, 2012). When boredom becomes too elevated, it not only threatens to turn into a lack of group motivation (Driskell et al., 2018) and risk-taking time-passing behavior such as alcohol use (Natani et al., 1970, 1973; Rothblum, 1990) but it also may hinder appropriate action in a case of emergency that demands to shift in a few seconds from a lethargic state of boredom into the highest level of vigilance (Rothblum, 1990). Feelings of boredom and monotony have regularly been related to a helplessness that encompasses a loss of control and agency over one's life and identity (e.g., Lugg, 2005; Eastwood et al., 2012; Raffaelli et al., 2018; Wolak and Johnson, 2021). As described by Eastwood et al. (2012) and quoted in Raffaelli et al. (2018), boredom can induce the sensation that one has “to do what they do not want to do or cannot do what they want to do” (Eastwood et al., 2012, p. 488). Similar conditions of boredom, monotony and hence a lack of agency have been reported in the day-to-day life of soldiers in Iraq where acceptance was the only manner to survive this “Groundhog Day” (Ender, 2012), i.e., acceptance of the conditions dictated by the environment.

Several interviewers described how the long-duration isolation narrowed their world to a tiny “goldfish bowl”, extremely magnifying the impact of daily events and news from the external world (Palinkas, 1992; Palinkas and Suedfeld, 2008; Driskell et al., 2018). Overall, all the interviewees reported to some extent how isolation impacted themselves, their emotion regulation, and the interpersonal dynamics. Retrospectively, most of them realized they were not able anymore to put certain things in perspective, showing again the importance of self-awareness, as discussed formerly with regard to sleep and fatigue. A well-known phenomenon of which crew members are also often unaware, and which has often been described in space flights missions (e.g., Kanas et al., 2001) and space simulations (e.g., MARS 500, SIRIUS, Supolkina et al., 2021), is the so-called detachment phenomenon (e.g., Kanas et al., 2001, 2007, 2009). The detachment phenomenon refers to the displacement or externalization of cumulated negative emotions toward the outwards on-ground mission control which has been interpreted as a consequence of increased sense of isolation (Kanas et al., 2001, 2007, 2009). Detachment goes together with a decrease in both positive and negative emotion expression (Supolkina et al., 2021) and may evolve toward a shared resistance in the crew against advice and decisions of the on-ground control (Myasnikov and Stepanova, 2000; Kanas and Manzey, 2008). Although detachment may create crew cohesion, it generates more distance toward the on-ground crew, carrying a risk for rash-considered decisions (Myasnikov and Stepanova, 2000; Kanas and Manzey, 2008). Similar processes could be observed in how the interviewees experienced communication with the external world. Whereas, all the interviewees ascribed communication difficulties with external friends and family mainly to the technical inconveniences due to the ICE-environment (i.e., asynchronous communication due to satellite connection with a small width band and subject to bad weather conditions), they attributed similar communication problems almost exclusively to the incompetency of the on-ground control crew. Moreover, in place of trying to solve dispute or miscommunications with the external organization, some crews started to gang up on them. Hence, detachment and the outwards displacement of negative emotions as a consequence of advanced isolation, should be an important part of a training program. Moreover—as suggested by one of the interviewees and in previous literature (Kanas et al., 2001)—these trainings should take a systemic approach, involving the on-ground crew in preparatory communication trainings. It is the on-ground crew with whom the crew will need to communicate during mission. They will be part of the mission, part of the system and thus part of the potential isolation and detachment effect they need to become aware of (Kanas et al., 2009). The hazard of non-awareness during isolation is beginning to be recognized. For instance, recent meta-analyses on teamwork in ICE-environments underlined the urge of implementing monitoring tools and cross-trainings to feedback crew members on their operational state (Landon et al., 2018; Palinkas and Suedfeld, 2021). Moreover, since the interviewees, in the current study showed some self-insight retrospectively, it would be interesting to compare retrospective data of mission experiences with data that were collected on location during mission.

On the level of group dynamics, the group showed a clear evolvement over time that corresponded with the stages described by Tuckman (1965). Getting to know one another (forming) was important as a base to be prepared for conflicts (storming) and to resolve them (norming) in order to reach good team performance (performing) and to be ready to finally split up again in a satisfying manner (adjourning). Although the storming stage is known to be a crucial and challenging stage for every group, it might even be more difficult for a group in long duration isolation. Time on station and how sojourners experience time in general, is a recognized important component of long duration missions. Time on station has been described to impact as to how sojourners experience their mood and motivation, pointing out that the second half of the mission would be more difficult and heavier to continue (Wood et al., 1999; Palinkas and Houseal, 2000; Kanas et al., 2001; Sandal, 2001; Supolkina et al., 2021). When halfway, one starts to realize how long the mission will still take; a low point that, in Antarctica, even may be worsened when it coincides with the period of continued darkness winter (Palinkas, 1992; Palinkas and Houseal, 2000; Palinkas and Suedfeld, 2008; Leon et al., 2011; Vanhove et al., 2015).

Most of the teams were mixed-gender teams which may raise questions on how somatosensory deprivation and loneliness may impact the interactions between men and women. Somatosensory or touch deprivation has recently been raised as one of the potential causes of emotion regulation problems and sleep problems in ICE-environments due to the craving of a specific population of skin afferents (i.e., C-tactile afferents) that are part of a psychophysiological regulation network (Van Puyvelde and Mairesse, 2022). To cope with somatosensory deprivation, one team leader explained inducing moments of physical contact which could be massage, a hug between crew members or self-touch. These moments were however strictly framed in beforehand to avoid any connotation or confusion with sexuality and were very well received by the crew. Indeed, an increased sensitivity of skin afferents may cause confusion between the experience of hunger for touch and hunger for sexuality. Hunger for touch is recognized as a primary need (Walker and McGlone, 2013; Floyd, 2019; van Raalte and Floyd, 2021) and can be satisfied both by sexual and non-sexual physical contact whereas hunger for sex can only be stilled by sexual contact. For example, the lack of a relationship between sexual desire and affect deprivation has been shown in a study, demonstrating that watching pornography when affectively deprived, augmented feelings of depression about the lack of affection (Hesse and Floyd, 2019). Moreover, an inadequate search for physical contact *via* sexuality can lead to romantic relationships for the wrong reason, unwanted attention, or in the worst-case, sexual harassment. For instance, Burns (2022) described how women have been labeled as “sexual hand-grenades” during some Antarctic missions and in a recent study on gender equality in Antarctica (Nash et al., 2019), 63% of the women received unwanted remarks from males during their mission. In the current interviews, only three out of ten women reported verbal harassment or unwanted flirtations, which is half of the percentage as reported in Nash et al. (2019). However, one could question whether the percentage in general is not higher since one female interviewee mentioned that unwanted remarks and verbal harassment still do occur regularly during Antarctic missions but that, on an official level, the management covers up facts. Obviously, to break the silence in such a small loyal community locked by the management about a topic that is sensitive and taboo, the responsibility to act should be taken away from the victim toward the entire network, inclusive the organization (Hershcovis et al., 2021). According to Hershcovis et al. (2021), on a preventive level, external organizations should invest in a gender-equal network composition (thus in the crew and the organization) and an educational change in certain belief systems. Some Antarctic programs report active efforts toward a more gender-balanced crew composition, however, they report difficulties in finding female applicants for technical positions, which ties in to a more general underrepresentation of females in trade careers. Hence, the most feasible solution could be found in increased investment in more gender-related training and a follow-up during and post-mission.

We suggest that the reason as to why the environmental factor is so overpowering has a lot to do with the fact that the impacted domains have a strong mutual reinforcing dynamic. Sleep, individual emotions, and interpersonal communication strongly relate to one another, underpinned by strategies of emotion regulation and adaptivity (Palmer and Alfano, 2017). On an individual level, sleep loss is linked with greater negative and fewer positive emotions (Zohar et al., 2005; Steptoe et al., 2008; Gordon and Chen, 2014). Moreover, sleep deprivation intervenes at every stage of the process of emotion regulation, i.e., the level of emotion identification, the level of the regulation strategy and the level of successful implementation of the selected strategy (Palmer and Alfano, 2017), which was also recently shown in a study at NASA's Human Exploration Research Analog (HERA) (Nasrini et al., 2020). On an interpersonal level, poor sleep is known to result in increased conflicts within couples and decreased conflict resolution (Gordon and Chen, 2014). Poor sleep was associated with a lower ratio of positive to negative affect and a decreased capacity to empathize and take the other's perspective during a discussion or conflict. To reach conflict resolution both partners had to be well rested. Importantly, the effects were not explained neither by individual factors of stress, anxiety, depression, nor by other relationship problems or the partner being the source of poor sleep (Gordon and Chen, 2014). Moreover, sleep loss also affects specific processes of frustration tolerance which is also crucial when living in conditions of confinement. For instance, poor sleep was associated with elevated negative affect in response to goal-disruptive events, but not in the absence of such events. Moreover, positive affect when reaching a goal was attenuated in situations of reduced sleep (Zohar et al., 2005). Secondly, insomnia research has shown the other direction of the sleep-individual-interpersonal (SII) triangulation dynamic, namely how daily problems may interfere with sleep. In these studies, it has regularly been shown that a lack of emotional and cognitive regulation results in ongoing rumination, impeding individuals to de-arouse which results in insomnia-related problems (Bonnet and Arand, 2010; Zhao et al., 2021). Thirdly, individual and interpersonal irritations have an influence on one another as well. For instance, in HERA, interpersonal conflicts predicted next-day individual strain and the other way around (Somaraju et al., 2022). Moreover, workload has a negative impact on this dynamic (Pfaff and McNeese, 2010; Somaraju et al., 2022), showing how the SII triangulation may interact at its turn with the work atmosphere on a station or during a mission and thus with the organizational level.

To combat the ICE-aspects of monotony, isolation, and confinement, most of the interviewees showed an extreme devotedness to their job (e.g., one interviewee who never skipped 1 day to go outside in the cold to control here device and data). Although we should take into account a potential bias in our participant sample (i.e., that the devoted people are also the persons interested to participate in a study post-mission), the presence of this feature supports some previous studies. Research showed that feelings of agency, self-esteem and usefulness are crucial to cope with monotony and, since people are cut-off from their normal sources to find these needs, they can find them in job performance and job satisfaction (Natani et al., 1973; Gunderson, 2012; Raffaelli et al., 2018; Temp et al., 2020). Furthermore, positivity was often mentioned as a strategy by the interviewees, which supports previous findings in ICE-teams (e.g., Kahn and Leon, 1994; Kanas et al., 2009; Botella et al., 2016; Palinkas and Suedfeld, 2021). By showing happiness, focusing on the positive aspects, and using humor, one individual may induce an emotional contagion on the entire group (Herrando and Constantinides, 2021). Moreover, it is well-known that positive feelings—also called salutogenic effects—evoked by the beauty and uniqueness of the expedition, the excitement, and natural grandeur are often experienced by people in ICE-sojourners as a counterbalance to compensate the difficulties of the environment (e.g., Palinkas, 2003; Palinkas and Suedfeld, 2021). According to Palinkas and Suedfeld (2021), salutogenic effects provide ICE-sojourners of an intrinsic motivation and optimism to improve interpersonal qualities and intrapersonal resilience despite the difficulties of the environment. Indeed, also in the current interviews—certainly in polar trekkers—these sudden intense feelings of happiness, uniqueness due to the beauty of nature were indicated as a motivational factor to remember why they had chosen to participate in the mission and to continue with new courage. Another recurrent strategy to combat monotony and isolation was to create structure, to plan activities and to stick to important dates. These are all coping strategies that have been described in previous work on ICE-environments (Suedfeld and Weiss, 2000; Smith et al., 2017). As stated by Smith et al. (2017), coping strategies are rather focused on managing and mitigating stressors than trying to avoid them. Indeed, the latter is literally impossible in ICE-environments. Leaving the station, a tent, or a spacecraft because one is irritated by other crew members may be impossible or lethal, as it almost happened according to one of our interviewers. Hence, a full comprehension of psychological team mechanisms in terms of team cognition, team cohesiveness and team motivation and how these mechanisms interact with the ICE-stressors is indispensable (Driskell et al., 2018). Finally, it was notable how a lot of interviewees described how they learned to reflect and behave in function of the group, putting aside their own individual perspective. It has been shown that a collectivistic approach in conflict situations will prioritize maintaining the relationship with others whereas individualistic thinkers will be concerned about achieving justice (Ohbuchi, 1999). Moreover, according to Triandis (2002), the situation in which persons are positioned can be a powerful predictor of the level of cooperation they will show toward a group. That is, culture is shaped by the environment. However, the other side of the coin is the risk to forget oneself and to lose oneself in the confined “goldfish bowl” culture of the station as was described in an Antarctic case study by Temp et al. (2020).

A large part of the interviewees indicated to lack of pre-mission training to prepare for their ICE-mission and particularly for the impact of isolation. When comparing the training programs that are currently established for space vs. Antarctica/space-analog sojourns, there is indeed a large discrepancy between both, showing a much more in-depth training for a very small number of people in the space part and a limited elaborated training for a large amount of people in the Antarctic part. Hence, to bring the best of both worlds together: the extensive experience of Antarctic programs in long duration ICE-missions could be combined with the conceptually driven approach of human behavior and performance programs from space agencies (as developed in e.g., Bessone et al., 2008a,b). However—as proposed by one of the interviewees—the question is whether one can be fully prepared after all and if there is a real need for a more appropriate training in ICE-missions. A review by Strangman et al. (2014) reported that they could not support, neither refute a negative impact of long duration space-(analog) flights on cognitive performance, which, at first sight, would speak against the need of training in both contexts. However, when looking more into detail, the story changes. Firstly, there is an undeniable methodological factor which may explain this zero-result. As proposed by the authors themselves (Strangman et al., 2014), all space-(analog) studies deal with small populations which obviously threatens statistical power. Moreover, in Antarctic deployments and non-Antarctic simulation studies, there is a large variability in terms of subjects, station characteristics and environmental characteristics causing interindividual variability both in terms of the subject and the mission (Strangman et al., 2014; Mairesse et al., 2019). However, the most important aspect in function of training is that space-flight subjects are highly trained astronauts with exceptional aptitudes and proficiency levels, which may demand much more sensitive measure methods than the usually used cognitive standard tests (Pattyn et al., 2009; Strangman et al., 2014). Indeed, this methodological problem of sensitivity has been identified by previous researchers as well. For instance, the paradigms used in some experiments have been argued to be too simple to reveal the subtle effects of space on performance (Fowler and Manzey, 2000). That is, for subjects who are overtrained to be as less sensitive as possible to the detrimental effects of spaceflight, the cognitive standard tests might not be demanding enough, which results rapidly in floor or ceiling effects. And while it is not detected by a cognitive standard test, astronauts may deplete their own cognitive reserve by doing more effort to maintain performance at an acceptable level (Strangman et al., 2014). This may also explain why astronauts in their subjective self-evaluation do report impairment in their flight performance (e.g., Bluth, 1984; Manzey et al., 1995), in cognitive workload (e.g., Burgess, 2000) and in fatigue (Eddy et al., 1998; Kelly et al., 2005). Secondly, there is a cultural factor that may play a role as well, namely how transparent a nation is in reporting deviations, errors, or other problems. An illustration can be found in the interviews and in previous literature with regard to gender-issues. Although gender-issues are still existing, they are seldom reported and/or researched. This may be problematic for both actual and future victims but also for the nations that do report, risking having the finger pointed at them, as if they are the only organizations having problems. A similar underreport is present regarding astronauts' performance when considering Western vs. Russian sources. First, published results from Western sources treat the study of performance as purely experimental: there are no available systematic reports on operational performance of astronauts. While Western reports of operational failures are merely anecdotal, describing errors in conducting experiments, in equipment handling or losses of experimental data (Manzey et al., 1995), several publications exist on Russian side analyzing cosmonauts' errors and behavioral problems. The “lack of official reports of behavioral disorders or significant performance decrements during spaceflights” described by Manzey et al. (1995) contrasts with reports from the Russian space program analyzing crew errors, being defined as a deviation from the standards of performance (Nechaev et al., 1998). This Russian error analysis confirms the above raised hypotheses based on methodological deductions, showing a measurable occurrence of real-life errors during spaceflight, with a rate of occurrence that mainly varies in function of adaptation processes, workload, and sleep/work schedules and that is highly dependent on mission/flight/task factors, population characteristics and individual operational features (Nechaev et al., 1998).

Hence, against this backdrop that highly trained space-operators show (latent) vulnerability for the impact of a space-environment (Manzey et al., 1995; Nechaev et al., 1998; Strangman et al., 2014; Gatti et al., 2022), that the interviewees in the current study experienced high levels of workload that may amplify the ICE-impact (Somaraju et al., 2022), that ICE-environments are considered space-analogs (e.g., Sandal et al., 2006; Palinkas and Suedfeld, 2008, 2021; Van Ombergen et al., 2017; Golden et al., 2018) and that (non-space) ICE-sojourners are generally rather inexperienced and naïve in operational safety, as shown in the interviews, we suggest that ICE-sojourners could benefit from space-based training programs. Obviously, this is not a straightforward easy task. Due to the above-mentioned inter-station variability and the heterogenous population on stations, a standard training must be tailored to the targeted ICE-environment. Moreover, the question remains at what point one is fully prepared, and which psychological (team) mechanisms (Driskell et al., 2018) should be targeted in a “good-enough” team training. Furthermore, as suggested in the current interviews and previous studies, these trainings should certainly include the different levels of the crew member's environment. That is, family members could be coached in interacting with their isolated relatives to be prepared for psychological reactions and crises and the entire crew-system (i.e., on-ground crew included) could participate in a training program on communication and potential detachment problems to safeguard operational safety (Kanas et al., 2009). Furthermore, the external organizations should be visible and transparent in issues such as gender, harassment, and mutual expectations between the crew, scientist and themselves. According to human factors models, a human error is the result of a systemic dynamic of interactions between a real time action and past latent conditions in the larger organization system and a failure on one level can impact events on the other level (Reason, 1990). In other words, human behavior and performance training should follow a systemic approach as has been recently described in a training program for elite Special Forces militaries (Pattyn et al., 2022).

The current study comprised some strengths and weaknesses. The studied sample was limited in size and unequally distributed over the different types of ICE-missions, with great variety in crew and individual characteristics such as crew size, station type, mission duration and environmental conditions. Although this was a direct reflection of the realistic distribution of personnel, it may have also impacted differences in the experience of the studied ICE-components. It should thus always be taken into account that the results reflect the ideas of a specific group of interviewees (Braun and Clarke, 2006). For instance, in our sample, isolation and confinement were often experienced as interrelated aspects and therefore also presented as such in the Section Results. However, for instance, if the sample would have included solo trekkers, the confinement factor would have been absent in certain experiences. Related to the latter, it would be interesting to compare, in future research, solo trekkers with confined populations to increase the insight in the particular contribution of each ICE-factor on a sojourner's experience. Despite of this inequality, we achieved to have an acceptable gender-balanced population (i.e., 14 males and 10 females). Moreover, both the similarities and variations that occurred over the different ICE-sojourners showed that ICE-factors are not static and in continuous interaction with the sojourners' varying adaptation capacities. A great strength of the current work, therefore, was the obtainment of first-hand information on ICE-experiences based on qualitative interviews. This approach unlocked new insights into where crew on these missions feel like further support, preparation, training could be provided. Moreover, the retrospective set-up, post-mission, offered the opportunity to the sojourners to take a step back and seeing the big picture again, underlining the importance of pre-, peri-, and post-data collection. Possibly as a consequence, some established aspects of ICE-life that have been described in former studies (e.g., changes in food taste, the impact of crowding during periods of crew change, Palinkas and Houseal, 2000; Vessel and Russo, 2015) were not mentioned by the interviewees and thus absent from the analyses. Finally, the systemic approach may have contributed to the agencies' quest for increasing knowledge about the nature of demands encountered by personnel at multiple levels of functioning.

To conclude, the interviews showed that the ICE-environment shapes the impact of organizational, interpersonal, and individual systems, hence influencing the function of sojourners. We identified station-related-, mission-related-, individual- and group aspects that confirms previous literature and that could be used to develop future ICE-training programs. Positivity, salutogenic effects, job dedication and collectivistic thinking were aspects that evolved spontaneously and sometimes compensated for the encountered difficulties, both in station and polar trek missions. We suggest systemically approached training, including all the involved parties of the crew system (e.g., family, on-ground crew) to better prepare future ICE-sojourners to cope with the impact of isolation and confinement. This training should be tailored to the specific characteristics of the station, mission, and group features and address gender-issues. Moreover, the risk of unawareness about the impact of poor sleep, fatigue and isolation on communication and operational safety should be highlighted for future sojourners. We recognize the difficulty to develop human behavior and performance trainings for such a diverse population, inherent to ICE-settings. Hence, the most pending question, remains, at what point one is fully prepared by a “good-enough” team training.

## Data availability statement

The datasets presented in this article are not readily available in accordance with the ethical procedures of ICH-GCP and GDPR. Requests to access the datasets should be directed to the corresponding author.

## Ethics statement

The studies involving human participants were reviewed and approved by the Commission for Medical Ethics of UZ Brussels. The patients/participants provided their written informed consent to participate in this study.

## Author contributions

MV, NP, and NS: writing. DG, NP, and MV: analysis. TV: data-collection. NS: expert knowledge. LB and SB-C: expert knowledge design and manuscript redaction. NP and TV: design. NP: supervision. All authors contributed to the article and approved the submitted version.
