# Supplementary material for: Living on the edge: How to prepare for it?
Source: Front Neuroergon. 2022 Dec 14;3:1007774. doi: 10.3389/fnrgo.2022.1007774 (PMC10790891; doi:10.3389/fnrgo.2022.1007774)
Supplement: Supplementary file 1 [file Table_1.pdf]

Supplementary Table 1. Environmental level. The E-factor.

**Monotony and harshness**	**Antarctica**
	"… of course, the monotony in Concordia is one of the biggest challenges that there is nothing at all is flat, there's no, no, colors outside, just the sun to create a little bit more color. Outside is even no cloud, there are no mountains, no animals, even no birds, there's nothing so any way it's really like being in one white sheet of paper."<br><br>"So actually, I never feel that sensation" … "Because every day was totally different for me. Mm hmm. And one thing was, I give credit to the cook that always always change the menu or keep preparing new things."<br><br>"I miss so much is one green space … I proposed the creation of one green space"<br><br>"… sometimes I was looking for, of course something new, you go outside, and you see black, white. Everywhere there is only white, white, white. And then every day if you are going outside, you see white, white, white."<br><br>"… like everything is all the same. It's all white, it's all always the same furniture, it's always the same smells the same people the same food."
	**MDRS/FMARS**
	"First time I went to MDRS, it was still quite new… and we got there after dark. And it was moon Full Moon, we just drove around the band around this big rock. And there was this white space station with glowing windows. And it was just a magical experience. And I think that has colored my my perception of it ever since … And, you know, you look out of the windows, and you look, you know, you can look out across the crater to the other side 22 kilometers away, and it's a Martian landscape."<br><br>"I really noticed that the colors play a massive role in how humans feel. So, in Utah, there's it's red color that surround us and you have sunlight which really benefits you. So, in Arctic we even though it was polar day, but the weather was not the best for us. So, we had only two days of sunshine. The rest was snow with rain."
	**HI-SEAS**
	"So, in the mornings, the clouds would creep into this, just this low in between, and the sunset over there as well. So used to get for the most spectacular sunsets through the window. But I mean, it also, it was just a load of red and black volcano. Volcanic material. Yeah. So, it wasn't particularly varied. But it changed enough that it was nice. Yeah. I thought it was a nice view. I would, I would say."

	**Polar treks**
	"Yeah, obviously, there are days like that. This is one of these days, where we just have to get on with it and just finish the day, and you just want the day to end, because nothing is happening. And it's a gale force, and you're just cold, so then I just look upon it as a job I have to do. I'm there for purpose, I'm going to the north pole or South Pole. So, it's just something that I have to stick with it. So that is the part of the job, then I just go into this rhythm and trying to get on with it actually, then then, sometimes it's important not to think too much. That's one of those days, when it's like that … one mantra would be, stick it out. Stick it out, just keep going"<br><br>"… then there is those days, you have to be proud of yourself. Well, that was a hard day that I did it. I went, I didn't stay in the tent, I did my 10 hours, on skis. I didn't manage to go as far as I hoped but I still managed to do that. So that, clap on your shoulder and you know, to be proud of what you're doing. I think that's important."<br><br>"…tried to divide the trip up according to important dates so at least I have something to look forward to which is not too far into the future … that can be you know, my son's birthday or other important dates."<br><br>"… just stupid little games. And if we were in a tent, like I remember tearing up a diary and making playing cards out of the diary, or we'd, you know, have little games that were trivia games or we'd, we'd talk about the end result. What are we gonna do when we get there? … What would you do if you're a secret agent? And or it could be real, you know, what would be your ideal marriage partner?"
**Changes in sensory stimulation and the sensory system.**	**Antarctica**
	"And then we have the darkness in the same period at Concordia. So, this doesn't help."<br><br>"And especially to not under-evaluate that kind of that… the winter. So, the three months of darkness, try to enjoy also this part because it's the real core of the winter because the three first months are still too much near the summer campaign. And the three last months also are near the other summer campaign. So, the real core is to enjoy these three months of that, even if there is no light, even if it's difficult, but it's also the part that after, people remember it most, most strong part of winter…"<br><br>"So, for example, one container or something with a few plants and some lamps because really the green is something that people, personally I missed that a lot."

	"But for example, I remember one day, when I was outside, I smelled the sea smell. It, probably is impossible because we were one thousand kilometers from. And I think maybe my body was looking for something, you know, different something. Not common, you know because you're going outside, that feel cold on your nose. And you just see white. And then one day you're going outside you feel cold on your nose, you see white but you smell something different."
	**Lunares**
	"I don't know if I mentioned that we were completely isolated from the sunlight. Which I think, I don't know if that's important, but I didn't mention that."<br><br>"The huge problem for example are eyes because you're always looking on the short direction so your eyes are not, your muscles are not exercising. So, you very often have this feeling that your vision is tired and everything what you can do is practising so I make them small exercises like they we all open all doors and we sitting on the one end and they have to I drawing something on the second end and they have to guess what I draw like…"<br><br>"It was, after, we were extremely delicate for noises, The Lunares hab is on the aiport so if somebody's landing or starting and you know there's this huge noise … there was already feeling something happened. And because we don't have like normally when you live in a big city you have a lot of extra noise, and we don't hear I think so 80% of that. In the hab, you are extremely delicate about the noises and … everybody's speaking so loud and everything … too intensive."
	**MDRS/HI-SEAS**
	"I brought some like … these scented oils. For for, for having a different smell, but actually, I rarely ever used them … I felt like they were kind of not sufficient. Because Okay, it's a, it's a different smell. But it's I mean, yeah … it's a certain artificial smell. It's not like fresh flowers or like some some grass or something."
**Physical activity**	**Antarctica**
	"there is one gym room where that is very, very, very important in the group because almost everyone would like to to participate on this sport. So there is one one bike, there is some tapis roulant, there is some weights and everyone at least one hour or even more try to spend that and I can say … this helped a lot"<br><br>"… a lot of people did a lot of sports and recreating activity like yoga. Like running on the treadmill and things like that. And it really helped a lot…"

	**MDRS/HI-SEAS**
	"But then finally, I made the connection that if I had moved a lot, I wouldn't have the back pain. And so that's when I started running on the treadmill more often … And then there was some I know from, from, other crewmates as well, that they, they, had like minor physical things that were mostly related to, either to moving much less or to just moving differently. Like I mean, you're sitting around much more often than in your normal work. You don't walk, you don't go to your supermarket … you don't really move much."<br><br>"So, we were mandated to exercise for an hour every day. That's what astronauts will do. So that's what we did. So, we all had about an hour of physical activity every day. Obviously, you could ramp that up to more if you wanted. Some people probably did a little bit less than that on some days. You know, we weren't perfect we you know, if you weren't feeling well, or something…"
	**Lunares**
	"Well sport sports kick them off to go on treadmill onto bike or whatever make yoga like really sports gave like … it's obvious that … gives you endorphins but especially in close environment"<br><br>"We have we have also a huge problem with space to make sport because in Lunares, treadmill is in storage room, so if you jogging in a small place without windows and without good aircon it start making sauna … in storage room…it is first not healthy, not safe and not make you feel better."
**Sleep and fatigue**	**Antarctica**
	"… and when during the night you, you don't sleep, it's very, very hard to wake up in the morning."<br><br>"… because the sleeping also is one of the biggest challenges in Concordia. Yeah, because of the pressure, because of the isolation, of course, but especially the lack of sun, the lack of production of melatonin. So, me for example I was bringing melatonin in Concordia."<br><br>"… we had bright lights either white or blue. And they were in the office. And you were supposed to keep them on from nine o'clock until about three o'clock in the afternoon. And, and they were to reset your circadian rhythm. And if you didn't have them on or if you weren't in the office to have them one, I found that I had a great deal of difficulty getting up in the morning … my rhythm sort of shift from say eight to 10 towards like a 10 to three in the morning or later."
	**MDRS/FMARS**

	“I had a dream one night about me and the girl, who had really, really lovely long hair long enough for her to sit on, and I actually dreamt that she got mould through her hair, and we had to we had to cut it all off.”<br><br>“I mean, I can't have ear plugs in the whole night. Like, every every day. Like I mean, I don't actually like wearing earplugs. But like when I was really really tired, occasionally … I would put earplugs in and I slept 11 hours and it was like heaven. Um, but yeah, apart from that I like I didn't try to take any medication or anything like that …”<br><br>“it's a bit hard to to say like, what, what's from lack of sleep, and what's from just being there. But I did feel that sometimes I had trouble focusing. Because I mean, if you if you're sleep deprived for a longer period of time, then I just have trouble focusing on something. Um, and I mean, I never felt like it was to the extent that I was becoming a safety hazard. But it definitely it's also just frustrating if you if you feel sleepy the whole time … I don't think like just sleep alone was really the problem, was just like the whole sum of things.”<br><br>“I mean, it depends on our workload, on stress. By the end of the mission, we had a lot of things to do, to finish. So, people were a bit stressing out, or they were thinking a lot before going to bed and then they couldn't sleep because of that. But yeah, I think sleep was better in Utah desert than in Arctic … Interesting thing that I remember one day, there was a strong wind in Utah desert, and I was sleeping in, in my dream I was I mean, kind of I was half asleep. And I thought that someone started to play music. I was like, why our crewmate is so crazy. It's like middle of the night, and he puts on the music. So, in … the morning asked, Hey, guys, who was doing that? And like nobody.”
	**HI-SEAS**
	“But sometimes, if you say one person Okay, go sleep. you have one hour extra for sleeping is like the best gift you can give somebody you don't have too much. But sleeping or resting is always the best gift.”
	**Lunares**
	“We didn't have a good solution, honestly. They were trying to, to play with our with our biological clocks with the UV light. You know, those those lamps that people in Sweden also use? And but it didn't feel like it helped. Maybe experiment was too short. I'm not sure. We we didn't have any good solution, honestly…<br>And we were, you know, with all the sleeping problems, if they try to, like give you more things to do. And we are telling them, we can't do more, because we're all like, lacking sleep, and it's very bad. And they are like, oh, but we have this and that … It was just very hard.”
	**Polar treks**

	"So whether that helps or not, I set an alarm and as soon as it goes off, I don't think I don't get into that it's really cold I don't want to get out of this warm sleeping bag, because then you don't want to get out and it makes it starts the day off in a very tough way. So, I don't let any thoughts in my head, the alarm goes off, and I physically react and get out. And then because it's cold, I just physically do what I have to do to get the tent warm, because I've got to warm my team and I think of my team, you know"<br><br>"I guess you will be tired, more tired after a bad night's sleep. But when you're tired, you tend to to, you get the rest you need anyway in the sleeping. But the worst thing is if you start to be cold and freeze, that's bad, because then you lose energy and you're shivering and you don't get to rest you need but even if you're kind of tossing around in your bag, and you're staying warm, you get you get rest through your muscles are resting. So, I tried to get eight hours of sleep every night in the tent. Or at least eight hours in the bag, trying to sleep. Important."
**Design and decoration**	**Antarctica**
	"So, what I really hate it is that there is no connection on the second or third floor between them. Instead, if you're at the third floor of one, and you need something from the third floor of the other you need to go, you know, down the stairs and then climb up again. And there's hypoxia. So, you suffer going down and up the stairs. And you have to do it, I don't know, like 30-40 times per day, right? Of course, this teaches you not to forget something on the other tower. But I think that this cannot be made because it will increase the wind resistance so much it would increase the risk for the station."<br><br>"And also other things that happen during the year or the winter then we we attach on the on the wall and so we we decorated also during the winter not not only the beginning…"<br><br>"… But having a window from time to time to look outside. We didn't have one, It was all under the ice… It got a bit better the year after because there was a project called the library in the ice from an artist from near Cologne, from here, not too far away, actually … and this library will be outside with windows … Sit down on a desk or on a couch and look outside. Yeah, so this is definitely something that I missed."
	**MDRS/FMARS**
	"And also, inside it was I mean, outside was greyish with a little bit of snow inside was also greyish. So, due to that, not everywhere we had electricity. So, there was no artificial lighting. And the carpets on the floor were also grey colour. Yeah. And so, this really starts to play with your mind."

	"It's noisy at HI-SEAS in the habitat. Now it's better from what I've heard, but when we were there, the sound insulation was really bad and so when someone was getting up, I would usually wake up. Some people were really quiet, and I wouldn't hear them. But some others were. Well, a bit clumsy. And then also, my room was exactly right next to the bathroom. And so, I would even hear when someone was flipping the light."<br><br>"I would add more ergonomics inside so, so it would be more convenient for humans to walk around more safe, like so we have this ladder from one first floor second floor … they said that they changed it … So also, like for safety reasons. You know, if you're in the middle of the night, you want to go downstairs to toilet."
	**Lunares**
	"I don't know if I mentioned that we were completely isolated from the sunlight. Which I think, I don't know if that's important … but I think not not being able to see anything outside was very strange, because it also looked very different from a normal home because we didn't have windows. Yeah."
**Technical restrictions: Asynchronous communication and internet issues**	**Antarctica**
	"Although it [internet] was slow, it was only 120 kilobits per second. I guess without the internet, it would have been harder. Yeah. Because it allows you to listen to proper radio, not just radio, on the radio. were, you just now have all the static noise and everything down there. But you could listen to the football on a Saturday. Yeah, you could listen to your … favourite music show … your favourite radio programme … You could read … the newspaper, you would have all information … like otherwise, before it was just"<br><br>"I can say I lived the past and the future of Concordia, the future that is even now. Because in the first two winters, we had only four times connection was possible only to send a few, we don't have internet for everyone, was only for IT, because was one dual out connection four times by day, sometimes was even out this lab connection. So, we was we had only possibility to send emails and to call 15 minutes by week."
	**MDRS/FMARS**
	"… sometimes if you if during the day, you consume too much of data, you've been almost cut off from internet, you have a bandwidth that is so low that it's difficult to open even Google on your navigator, your web browser [MDRS]… For FMARS the situation was a bit of unknown because the communication system relies on satellite communication for telephone and internet. And none of us had previous experience there. So we didn't know anything of what we should expect during FMARS. I told them, well, I don't know if we will be able to communicate, communicate with you for the during the mission. So if not, don't panic, go to the mission website and see if we are still alive"
	**HI-SEAS**

	"And for us, we had the asynchronous communication, so it was really hard to mail. So, a lot of the things of like wanting more information during training about how hard that is that I was not aware of before and so that would really drive me low…"
**Potential life-threatening due technical issues**	**Antarctica**
	"And during that most cold, most cold part of winter, when it was necessary that they work the most, this engine was broken in one every week. So, we arrived at the month of, was one month before the end of the campaign, that we had only one security group, and one in one of the groups of the power station. So, this situation was very challenging … and created a lot of tension in the group because, of course, if this case up and was, was really the end because everyone will be frozen. In that case, was T -70 degrees outside … Finally, they discovered that it was because of the oil that they were using … it was necessary that some people checked one level of the oil in these motors and everyone in the group participate in this task … to avoid that the oil exit from this machine. So, I remember that the IT group put some cameras, and everyone from his own laptop two hours by day or one hour by day should monitor this this camera…"<br><br>"o yeah, we had only one blackout. That's incredible. I think it's the first year that only once happened. And it lasts for maybe five minutes not more but five minutes without electricity seems like five days there, it's really it's really scary. And yeah, other big problems."<br><br>"If you cut yourself in Antarctica, you don't have the the the blood in the in the refrigerator and then there is the machine that gives give you the chance to have a transfusion of blood from one person to the another directly but it was not working first."
	**MDRS**
	"At MDRS, water was brought by someone outside on a trailer. And there were a couple of times when the weather … because of the weather, the road was impassable. And we were starting to sort of you know, within a day or two, running out of water. So that was a bit of a stressful thing. We never did. But that was a factor."
	**HI-SEAS**
	"And we got an email one evening saying the water trucks are coming because we had to we had to prepare, we had to cover the windows and stuff so that we wouldn't break isolation. And so we took that to mean water is coming but what was meant was that the water trucks are going to test the road to see if they're willing to drive up it. We went and abused a whole bunch of our reserves. Because we thought we didn't need it anymore. And then unfortunately, we did that in the time that it took for someone else to notice and to clarify that water is not coming. So that in itself, it was just a miscommunication."
