# Supplementary material for: Living on the edge: How to prepare for it?
Source: Front Neuroergon. 2022 Dec 14;3:1007774. doi: 10.3389/fnrgo.2022.1007774 (PMC10790891; doi:10.3389/fnrgo.2022.1007774)
Supplement: Supplementary file 2 [file Table_2.pdf]

Supplementary Table 2. Environmental level. The IC-factor.	
**Loneliness, home sickness and contact with the external world**	**Antarctica**
	"You have to consider that on this specific thing I had two extreme people: X that always wanted to communicate with his wife every day at three o'clock in the night ... And Y that called his mom, so his family just two times. I'm not joking …"<br><br>"… but at the same time, we face … the loneliness, because there are other people there, but they are foreigners. And then you have, eah, with them sharing an experience. But at the end, you don't know them, and they don't know you ... in some way they are foreigners and then, eah, you are looking for … your life outside. I remember when we had the chance to use the VOIP phone ... I just took the phone… and called somebody, even if I didn't hear from a lot of times, and they were very happy to hear me from that part of the world. But, also, for me … was something to help me to feel less alone, let's say."<br><br>"My family gave me an assortment of small parcels to open at set of times during the winter. So, if I was feeling a bit down or if I was, it was just a long week, then I could open a parcel and there'd be something."
	**HI-SEAS**
	"Like, they're really excited with this research also, it's like getting a lot of publicity through the media through being here at HI-SEAS. You just felt like personal accomplishments outside of the mission itself. So, I think that was very much a motivating factor."<br><br>"It can be a little overwhelming, you're away from friends and family … We didn't really receive much training regards that, and I think the purpose of that was they didn't want to alter, you know, our, the results as well as what they thought may be important when it came to how the crews would prepare on their own."
	**Lunares**
	"So, we hugged ourselves if something happened. If somebody had a problem with the with the hand, we massage this person so there was no problem with and there was completely unsexual touch. But we constantly like you know, in Japan, you have this one metre. And if you touch somebody you're over. Yeah. And that's very interesting because I wrote a whole chapter in my PhD about how important is the sense of touch in the human like to stay healthy mental health … I think so it change something."
	**Polar treks**
	"… in the first, the first trip, there was no contact with home. And, well, I don't know If it is, it's kind of, we call this all sides of backside to be able to call back home all the time because suddenly you're pulled away from the situation you're in, you're home, mentally, you're talking to your wife or children. It can be tough

	actually to have that when you are in that fragile field operation. When you have a bad day and you're talking to your your five year old and things like that and he is actually asking questions like why, why are you here, you should be home and things like that. In one way, it was easier when there was no satellite phone and there was no possibility to call back home at all. So you would just stay in a good mood, in a stable kind of mental situation all the time. So, in one way that was easier."<br><br>"You're having your breakfast, that's when your emotions and your thoughts and you're what am I doing, is this worth it, it's terrible… If you've got one of those days. And again, for me, it's quite, I am quite a logistical or a pragmatic person. So even if I'm thinking, I miss home, I'm tired, I will think I am here. It's an honour to be here. So I , it's how I get through all the time you have an honour to be here, you've left people behind your family who are trusting on you. There are people that have sponsored you, there are people out there that you've got to service, there are people relying on you. And it's okay to fail. But you can't say I gave in, you took this job, nobody asked you to do it, you took it. So you have a duty."
**Goldfish bowl effect**	**Antarctica**
	"it's not a very big important thing, is not useful to say outside: ah you know I have these problems because this is a stupid thing that you can have also in a normal life. But when you are far away, it's it becomes bigger and bigger than it was. So normally I always keep everything inside Concordia."<br><br>"I guess when people are in isolation, so not in a very calm state of mind, it's not so easy to to evoke the rules that you have agreed on before. So that was lost … And, of course, things get aggravated when you're in isolation, much more than the real problem is."<br><br>"It was about, especially about the behaviour during the isolation, was some tips for example, to what can happen with the people can become more sensitive during more time they spend in Concordia, the more they become sensitive, even to things that in normal life usually are not important. So, for me, I keep really this information very seriously. And even I was repeating myself when I was in the situation of stress and some something what was happening and I was starting to feel the stress and and in difficult situation, so I repeat myself this is because of the isolation is not because this person is particularly bad. So, keeping this in mind, this was really very helpful for me."<br><br>"… because of the isolation, as I said before, and sometimes the sensation becomes so extreme, that people really think that the other person is one very terrible enemy. In some cases … some people stopped to talk to each other forever, so was extreme this this conflict they had."

	"But then if you're able to just look at the bigger picture, you realise that actually, there's nothing wrong with what you've been doing. And it's completely unrelated to the actual sort of problem. And the problem is something completely different."<br><br>"… getting people's perspective back, you know, I found that in a small group environment, often very small things get blown into almost paranoia, and people lose their perspective … But there just were occasional moments when I really felt that I needed that connection to my past life to the outside world. So I remember once, I was quite upset, you know there had been a big bust up on base. And I just felt like I totally messed it up, I was a complete failure, I was really down about it. And I rang my best friend from home, on the satellite phone, probably the most expensive phone conversation I'll ever have in my life, because we spoke for ages. But it just, you know, totally revolutionised how I was thinking and how I was feeling because … I was able to to put things into perspective…"<br><br>"… who had a coffee mug that he didn't want wash, and somebody new came into the base and didn't know that and they washed his coffee mug ... thinking they were being nice … I've never seen someone just go from completely happy to total, like devastation and fury, all wrapped up in the same package."<br><br>"I mean you start off thinking it's gonna be forever. And suddenly, you've only got a couple of months left. And you think oh … I wanted to do so much of this and so much of that and then suddenly the planes arrive and then suddenly you think oh, they're here and then you think oh, then I've got lots of time to enjoy, like the sunshine and then suddenly you're on the boat leaving and you think oh when did that happen, what happened to the last year and a half, and then you come home and you think. And that was a bit of a strange thing, I go away and do all this stuff in the middle of nowhere and it's a big experience for me and I come back and then everyone else is just 18 months older and nothing else has changed."
	**MDRS**
	"… one crewmate was yelling at me for something I was, I don't even remember. It was complete bullshit … And so I was like, Did I do something wrong? And then she hadn't even realised that she was like doing this ... And, I mean, that was just not her style. And it was just not her. And usually, we got along really well. So that was like, the trigger for me, okay, something has to change drastically, because this isn't like we have several more months to go. And if we keep going like this, we are going to come out and have PTSD."<br><br>"… sense of humour. That will help a lot in isolations."

	**HI-SEAS**
	“bigger picture, not forgetting the bigger picture and, and discussing the bigger picture…”<br><br>“As far as conflict goes, the little things that annoy me did seem bigger. I am very sensitive sometimes to annoying sounds so someone is like rattling something or chewing loudly it can really annoy me and that happened a lot more often there, partly because it's quiet and partly just because I’m stressed and paranoid.”
	**Lunares**
	“Yeah, because there everything is amplified, everything… everything what you hide in isolation will blow up and blow up very hardly so especially when you have one problem with the mental problems already like without sleep or without eating. So, … I had this feeling that I'm talking almost all fucking days about problems. And sometimes was like extremely stupid problems. You know, he hung his towel on my bag…”
**The importance of food**	**Antarctica**
	“… we had some someone who hided loads of food in the roof, which I thought was an interesting thing psychologically … although we have loads of food at Concordia and you never really like run out of … but it was kind of I think people really struggle with like losing, losing that control over their lives.”<br><br>“… the people that is in charge of buy food for somebody that are going to do the winter, have never been in Antarctica for the winter. And he's not focused on give to the people something … to rely on”<br><br>“we had a cook, who was pretty good … like he was very good at producing suddenly, a plate of chocolate eclairs … and it'd be like the best day, cause you’d have chocolate eclairs all day.<br><br>“… So it was miserable. And it had such a clear effect on everybody.”
	**HI-SEAS**
	“… that was one of the things like if we did have really bad coffee that made us feel like we were miserable. Even if we didn't have good sleep, as long as we had good coffee. Because we knew like which coffees we wanted, and they might want to give us like … we wanted to like kill somebody.”
	**Lunares**
	“everything was artificial, light was artificial, air was artificial, food was fucking artificial…”<br><br>“and then I realised that if the food is boring, I'm having a bad day…”
**Spending time**	**Antarctica**

	“We always watched Game of Thrones, or some kind of series in the evening … And I think it was about having that kind of routine…”<br><br>“We regularly had Saturday night dinners … everybody dressed up for them, everybody came … And it was a real moment where you kind of all came together as a group and had a great time, I think that was really beneficial in developing a sense of group cohesion … so, there was once a week where everybody gathered.”<br><br>“I just I just said I just I'm gonna be in my room now for a day because I just needed silence … I just can't because it was just my brain had to be put in order again. It was too much influx.”
	**MDRS/FMARS**
	“one thing that really helped me and I also got the feedback from the crew that was helping a lot so what we did there was we had breakfast, lunch and dinner together, not just not only dinner, but all the meals together. And then before before breakfast and before dinner, we would often not always but often do exercises together. Just very simple exercises. I mean 20 minutes of either yoga or something more … It was just doing something together.”<br><br>“I was completely unable to tell, like what day of the mission it was. And if I hadn't had the calendar on my computer, I wouldn't have been able to say which month it was.”<br><br>“And I had no trouble to go into my bedroom like very small bedroom to isolate myself and that didn't pose any problems with the crew members or the group dynamics and each of the crew members did the same at some point. So, I guess we had to balance between, you know, group activities and some time and alone time.”<br><br>“I know that that's in some simulations, they didn't eat together, everyone was eating when they want, like separately, which is something … meals together really brings the connection. And it really helps to have this family atmosphere. So, we always had this in three months, in one month in Arctic, like breakfast, lunch, and dinners, always together, or, like cooking together. Also, cooking is really good and fun way to, from, you know, shake off the stress and have the jokes.”
	**HI-SEAS**
	“I think there were times where when I wanted to just be alone, but I know probably around the crew, I would like to just put headphones on as I continue my, you know, my paper write up for my personal research. Or

	just I would just play a video game on my personal computer ... When there were times where I wanted to just be on my own, like, I'd go up to my room, and bring my computer with me and do work up there."<br><br>"I took like 200 pairs of earplugs, I guess. Or just a ton of them. Yeah ... And if there was too much noise, just stick ear plugs in, or put music on … Maybe made me able to isolate myself better was the key thing. So, if I really wanted some peace and quiet in my room to write or do something, I could just go up there, stick some earplugs it in … I would have been a terrible crew member if I hadn't had them, I don't know."
	**Lunares**
	"We were trying every day to have every meal together. And that was very important. So, for example, when somebody was running late, because we had some unscheduled, whatever activity … everybody would wait, even if we were hungry, everybody would wait for the last crew member to arrive. I think the meals were the most important part of the day, honestly."
