# Supplementary material for: Living on the edge: How to prepare for it?
Source: Front Neuroergon. 2022 Dec 14;3:1007774. doi: 10.3389/fnrgo.2022.1007774 (PMC10790891; doi:10.3389/fnrgo.2022.1007774)
Supplement: Supplementary file 3 [file Table_3.pdf]

| Supplementary Table 3. Organizational level | |
|---|---|
| **Conflicts, Irritations versus loyalty towards the External Organization** | **Antarctica** |
| | "… who is here in Europe can't understand what is really happening … eah … And no one has the right to tell something from the Europe … some moments of frustration … mainly due to organizational problem. So, everything external to the team …"<br><br>"… one thing that I miss is when you're going in Antarctica, there are a lot of health check. You know, there is two days of a check … when you come back, nobody asked me anything or just again tell you to in one month go on this hospital and you will have a check and I think this is not good. I mean you spent one year there for work and it is like respect of the work, you know because of naturally there are a lot of particles going on your body because it's a very high altitude."<br><br>"… you don't know anything, you cannot do anything, you are scientists, you are the worst …"<br><br>"…once again, picking the station leader, the technical Chief, and all these roles. This is done by administration, … And this in my opinion, it could be improved. And, also, it could really depend on who is the character that is fit to do it. And not really, in having a balance between nationalities and all that." |
| | **MDRS/MARS500** |
| | "I assume that instead of making sure that the crew members would be on the same page before the mission, they also should do the same with the remote team"<br><br>"… the communication with the outside was mainly with the duty team and was changing all the time. They were our point of contact, but then we had potential different persons we would talk to, and some persons had more impact on us, for example, the person in charge of planning would send us every week a planning with what which experiment, we need to do when … And sometimes it just didn't make sense. And we're all wondering inside, why, I just don't understand, it is just painful to do in this way ..." |
| | **HI-SEAS** |
| | "… we couldn't usually get the information we needed from mission support. And I think part of that is a reality of the staff of volunteers. … what we should do is get a lot more money…"<br><br>"… he managed to eat, but he just didn't manage to eat in half an hour … you know, it's those kind of things that get overlooked that some certain things take longer time. And in the beginning, we were often, not often, we were always running behind schedule." |

| | |
|---|---|
| | … maybe everybody could have used a little bit of training in how to be helpful on a delay. Like, with a 4h shift, especially when the crew is working during those four hours, it's really hard for to have a back and forth you need to do any clarification on a question [by written mails] … once I said, like, I think it was can you, can you get me a recording of X, and the person responded saying video or audio? When I got it, I was like, I don't care either, both. But by that by the time I got a response, it was on to the next person."<br><br>"And especially there's some trust that's required because I think … I know he's better at finding stuff on the internet than I am. He's better at getting stuff done than I am. So, if he comes back, and he needs clarification or can't figure something out, then I know there's a real issue, like I wasn't clear, or it can't be done. Whereas with strangers, you wouldn't be sure like there could be incompetence."<br><br>"Though, when you need something resolving you need an answer, they'll say, I don't know and try to direct you to someone else. And then it's easy to get lost in this 4-person leadership" |
| | **Polar treks** |
| | "Because we wanted to help scientists and we were working with scientists around the world. And that was what gave us the, if you will, the motivation to keep going when it was really bad … it's just hardcore data … then you've got more chance of putting your story out there of the difficulties that the Arctic's facing…"<br><br>"… how I get through all the time you have an honour to be here, you've left people behind your family who are trusting on you. There are people that have sponsored you, there are people out there that you've got to service, there are people relying on you. And it's okay to fail. But you can't say I gave in, you took this job, nobody asked you to do it, you took it. So you have a duty." |
| **Security** | **Antarctica** |
| | "For example, for me, it was interesting that there was no proper inventory of medication. With expiration date, what do we do, or on the other side, a log of what has been used, so you can order the proper stuff for the next year. Yeah. But we worked a little bit with the other doctor on that, and hopefully, I think they took it up. Another thing that now I know has been fixed is telemedicine … In theory, we had a system in place. So, there was an operating room, an operating theatre, if you will … And then there was supposed to be internet connection to some specialists in a hospital in Italy that could guide you through because none of us was a surgeon. Yeah. But we never had the chance to properly test that, despite our inquiries. And so of course, this makes you feel insecure, because what do you do if you really have to do it?" |

| | |
|---|---|
| | **MDRS/FMARS** |
| | "For instance, we we, arrived at MDRS … and we asked for first first aid kit for like a month before we got got it and we had several minor injuries during that timeframe. And we couldn't properly address the situation because we hadn't the medical kit we should had."<br><br>"Sometimes I get to hear about simulations or even see simulations that are starting from scratch and they're making the same mistakes that have been made so many times instead of learning from the things that have already been found" |
| **Preparatory Training: Training** | **Antarctica** |
| | "But you know, a lot of things that happen on base, either are difficult to put into a formal training or formal advice … so perhaps there's a lot to be said for just sitting and having in an informal setting, having a relaxed chat with someone who's already done it and yeah, hearing their experiences and, and then…"<br><br>"… it helped already bonding the group together … was not so much the skills themselves more with what happened during this week, and probably the same for the firefighting course. Because … both are somewhat extreme situations. So, this is definitely the one side of preparing you really to go there and to be prepared for a situation that might arise, but also would bond the group."<br><br>"It was … especially about the behaviour during the isolation, was some tips for example, to what can happen with the people, can become more sensitive … more time they spend in [isolation], the more they become sensitive, even to things that in normal life usually are not important. So, for me, I keep really this information very seriously. And even I was repeating myself when I was in the situation of stress and some something what was happening and I was starting to feel the stress and, and, in difficult situation, so I repeat myself this is because of the isolation is not because this person is particularly bad. So, to keep in mind this was really very helpful for me." |
| | **MDRS/FMARS** |
| | "So, among these people, we were kind of, we had a good vision on what we should expect for the future mission and what we should do there. But it doesn't, didn't include all the the remote team that was that that's also a big part of the mission, you know, because we mostly communicate with them when we have a particular issue, technical issue or stuff like that. And and these people were not prepared to, to make this mission happen." |

| | |
|---|---|
| | “You know, well, I think people should be warned … And we were also thinking of writing instructions, psychological instructions, for one for people who goes to isolation. So, to mention the third quarter phenomena. So, people would be aware, if all of a sudden in the middle of their missions, they feel quite down. So, then they would, you know, again, open this instructions and read about that and look, okay, and I'm fine is just, you know, this period, and blah, blah. Also, there are many other phenomena that happens in isolations. And I think people should be aware of that. So they wouldn't think, Oh, I'm going crazy, or something is wrong with me. I shouldn't do that. And I shouldn't feel that. So, they would put even more pressure on themselves. They this will be very helpful. I think this this will improve their well being. Because if you're aware of what's going on, you're you can be, you know, prepared for that” |
| | **HI-SEAS** |
| | “I definitely would have been happier with more training. I felt both confident and not confident. I think I understood some of the details of the habitat and my life day to day pretty well, like what kind of cooking facility we had, what the layout of the habitat was, which helped me feel confident, but I don't think I was well prepared for just how hard it is to keep in touch with family members. That was really difficult, and I don’t think I was well prepared for just some of the feelings of, you know, extra stress, paranoia, the isolation effect of that site. I was warned, but maybe not warned enough, I guess.”<br><br>“We didn't receive a lot [of information]. Before the mission started, we had Skype discussions with some of the organisers, and they sent us some information, both just verbally and also some pamphlets or like a document, their general pieces of information were statements, you know, about what to expect that, you know, that can be hard that, you know, some of the challenges we might face, like conflict, like there will be conflict, inevitably. So, we're looking for solutions to that, you know, don't expect you won't have any conflict. Once we arrived in Hawaii, before the mission, we had about a week of training, and we received a lot more there, both technical and kind of psychological. And that was also very kind of general advice, some general notes about past things they've seen, you know, remaining anonymous, but the idea that things happen fast. That was probably the biggest piece of advice that you get. You get angry very quickly you might become very happy quickly, much more than in regular life.”<br><br>“So when those issues did arise, how people would handle them, and how they would want to handle them both individually, but also, as a crew, you know, how they would want to talk with other members potentially. That was probably some of the major trainings that I've had prior to HI-SEAS, and it was very effective and important before going into the mission itself.” |
| | **Lunares** |

| | |
|---|---|
| | Responding to your question: “So there was no formal training” “About soft skills or something? No.” … “No, I was honestly not happy with the way they prepared us. Not with and also not with the way they chose us. Because there was a selection, and yeah, but I think they didn't see some problems coming.”<br><br>“They were organising some more technical trainings, but not all of them were mandatory. So there are crew members who bonded more with smaller teams like 3 people or whatever. And there were crew members who knew each other beforehand ... So I think they should have put more effort to try to familiarise everybody with everybody, if you know what I mean.”<br><br>“… this whole like mission book, which we had was very specific about technology about you know, everything what we have to do, like you can see that engineering draw this whole sheet up. But there is nothing about the real human beings.”<br><br>“... I think so this is the huge job and I'm not sure that this … really… like the people from the organisation are already thinking about this seriously, that preparing the team is the success of the team.” |
| **Preparatory Training: Inter-crew Changes** | **Antarctica** |
| | “Ideally, I think we should have some material already set out for every age group or gender or role. And then if you would have someone with the experience, it would be good to hear from them … Maybe if I talked to someone that was 60. His advice would be different. Might have worked for me might not have worked… but also dedicate some part of it to have personalised coaching, at least for beginners. If I have never been to an isolation mission before, maybe I would need some advice. Personally, for me, what does that mean? For my role, for my age for my gender? So you know, because if I'm male or female, I may have to take care of different things in behaviour in preparation, if I am 25 or 55, again, my relationship with others will be very different. So, what do I have to take care of?”<br><br>“… probably those things that you wouldn't have written down anyway. Those things how, what these stories they tell you, so it's more like that you learn from the storytelling… this might have happened, and we acted in this and this way or … Now that there was a like eah, now we laugh about it, but then this was pretty scary. And this is what's happened. So, by this, and if you get into the situation, look out for this, and that this would be probably like, you know, 3000 years ago, a tribe round the fire, probably closer to this.”<br><br>“It might be good somehow for the crew going down to, to be more in touch with previous crews, I think. And I don't know, like a kind of buddy system” |

| | |
|---|---|
| | "And this was a surprise to me, too. I think it was someone in my family before leaving … they gave me a plastic little tree. Yeah, fake tree, of course, because you cannot take real plants in Antarctica, or anywhere else. But I thought it was kind of, you know, I mean, why do you bother giving me this, it takes so much space, blah, blah, blah. And in the end, honestly, it was the most wonderful thing in my room, just seeing it there." |
| | **HI-SEAS** |
| | "… I kind of avoided it because I didn't really want to see other people's experience. I wanted it to be fresh. I didn't look at crewmember information very detailed." |
| **Preparatory Training: Preparation of Family and Friends** | **Antarctica** |
| | "Yeah, first of all the booklets … that ESA printed. I had them skim through it or read it. And I think there is a page right, correct me if I'm wrong. For relatives or people on the outside"<br><br>"I could just relate to them. What I was told from the one from from the Institute. Yeah, so, I would, I would tell them stories so … their questions would be similar to my questions. Yeah."<br><br>"I have really good memory about the moment I mean, when when he got this news … We stayed together, we we cried together, and we hugged each other. And it was a really strong sharing moment. You know, from the moment we started to say closer, even physically. Because we felt that being close, it was easier for us to manage the situation." |
| | **MDRS/FMARS** |
| | "And none of us had previous experience there. So, we didn't know anything of what we should expect during FMARS. So I was, I told them, well, I don't know if we will be able to communicate, communicate with you for the during the mission. So if not, don't panic, go to the mission website and see if we are still alive."<br><br>"I think that you when you have this limited internet, or limited time to talk to your, relatives, so the way you you write one big long letter, and you start to learn how to write those letters, because nowadays, we just, you know, do this messaging as short as we can. But there you really write like an essay, about your feelings, how everything is going, and then you don't and you can, forgetting that people outside of simulations, they have busy lives, and they can't reply you straight away on at the same day, or even in, you know, few days. So, I would just recommend not to get crazy. Oh, if they forgot me why they don't reply to me straightaway, and etc, just to wait a few days, maybe even a week? And then you know, yeah, you can get I think this frustration, even if you get the reply, and it's only one sentence, then you're like what I wrote so much. Yeah, |

| | |
|---|---|
| | that can happen so maybe before you go to isolation, talk to your relatives and friends, and also discuss this, like that, you know, it will be different. And I would appreciate if you reply and reply, just, you know, a bit more than in usual day.” |
| | **HI-SEAS** |
| | “I don't think I was well prepared for just how hard it is to keep in touch with family members … That was really difficult … Number one, for sure was outside news. If I got a letter, an email from someone that was unhappy or frustrated … And that would just destroy me for like a whole day….”<br><br>“… we had the asynchronous communication, so it was really hard to mail. So, a lot of the things of like wanting more information during training about how hard that is that I was not aware of before and so that would really drive me low. Most of the stuff in the mission, I felt like I could kind of shrug off sometimes… Emotionally wise, I would recommend that they establish some needs and wants from their loved ones and let their loved ones establish needs and wants back so that they have a better plan in place for how to deal with emotional difficulties. So, if someone will need certain kinds of communication or they're not sure, promise each other to check that out again, during the mission and check in on each other, because you have to be a lot more diligent than you do in normal life.” |
