# Supplementary material for: Living on the edge: How to prepare for it?
Source: Front Neuroergon. 2022 Dec 14;3:1007774. doi: 10.3389/fnrgo.2022.1007774 (PMC10790891; doi:10.3389/fnrgo.2022.1007774)
Supplement: Supplementary file 4 [file Table_4.pdf]

Supplementary Table 4. Interpersonal level.	
**Team Dynamics over Time: Getting to know during Crew Selection and Team Building**	**Antarctica**
	So we, from early on, start talking … what could we do together and implement in our daily life, so to, to function better together to, to make common experiences and, and from this many, many things came so that we always could say, that we would at least have two meals a day together? Yeah. That we would do different sporting, or celebrate sporting events? Yes. That we always would pay attention to, for example, a crew member's birthday…"
	**MDRS**
	"And then they selected the best crew, the best crew, and some modification because, well, two years passed between the 2 weeks training mission … and the start of the … programme."
	**Lunares**
	"So yeah, I would say that was a dry run, there was not really like supervisor for that. Taking care of how we interact, we just figured out how to be around each other."<br><br>"I would advise them to try to know the crew before they go. And if that's possible, to find some hobbies, or even topics to talk about with your crew members."
	**Polar treks**
	"And the other thing, which is important when it goes to choosing a partner, we need to both have the same motivation and mental capacity because some of these trips are just so hard, like the winter trip to the North Pole I did … it's just so hard and you can't do the expedition mentally for two people, you have to know that your partner is able to take care of himself and he's motivated."
**Team Dynamics over Time: From Conflicts and Frustrations towards Conflict Resolution and Teamwork**	**Antarctica**
	"… we had a set of rules, but we did not necessarily stick to those rules. I guess when people are in isolation, so not in a very calm state of mind, it's not so easy to to evoke the rules that you have agreed on before. So that was lost. But we did have conflicts."<br><br>"… one conflict between two persons and already all the group can be broken … also support some people during one conflict is potentially dangerous for the person that supports the person … the person that was going to try to give help was target, like, also enemies of this other. So yes, this is very often, it's enough one conflict and the whole group is already broken."

	"… it's very very, very difficult because there is also … external reason can be some trouble at home, can be a brother or a sister that have some sickness or even in some extreme cases, some parent that die I also experienced something like this during one of the winters one member of crew lost a mother…"<br><br>"And then the first big change in the dynamic is when the handover. So, the old team might be still there apart from your team, but you're now in charge, it's now your base. So that's first time where you then you start to make your own rules, also your own rules that might impact other people on station. So, you might say from now on we have these and these rules for the summer days like when to clean the station"<br><br>"And in that case, also some people that was not speaking each other, but at least they shared the task, they were in the same group of four for example, and in the end was very positive. In many cases, it was possible to see the group again or compact during these special occasions. I see also the same when was necessary, for example, to work to remove the snow from outside before the start of the summer campaign was some common task for the cleaning of the base, for example, where everyone should clean one, one part. So in that case, The work and the duties was helping to keep the cohesion of the group so, this I think yes I noticed Very often."
	**MDRS**
	"I can only think of one occasion at Utah where we were sort of arguing or discussing forcefully. And that was over. It was interesting, it was not something internal. It was something external. It was how to interpret a particular set of instructions for Mission Control. Yeah"<br><br>"One thing that helped a lot was having a crew member who just cracks jokes, even at very inappropriate times. You know, nothing's better you know, like everyone is like fighting, like having a big argument. And then someone just says something totally stupid, and you can't help but laugh that just releases the tension. So that is extremely helpful. I mean, it doesn't really solve problems. But it helps relieving the space for a moment and like, sometimes, like usually when, when that happened, it gave the opportunity for everyone to just, you know, mentally take a step back and think about Okay, what are we actually fighting about? What are we actually arguing about? So that was extremely helpful."
	**HI-SEAS**
	"I mean, if you stick six people in isolation for eight months, there's gonna be a conflict. So yeah, we certainly had our conflicts. But I think the way that we dealt with them was really good. We, at no point did anyone raise their voice or yell or scream or anything like that. And I thought that was, in reflection, it was pretty surprising."

	"… most important things there was that we were all very committed to crew cohesion … we decided as a crew what, what we cared about and what we wanted … And we decided to prioritise crew cohesion and good data."<br><br>"The power of one I think is important, when you realise that it takes one person to stop and reverse the mood and some problem that's going on, and being the first person to say, hey, this thing bothers me. … When you realise that you have that power as an individual, it gives you a sense of control, that you can actually change the thing and whatever is annoying you."
	**Lunares**
	"But on the other hand, that was very clear that they had the biggest conflict between each other. So at some point, me and the other female, we were really trying to calm down all the rest. And it worked to some extent."<br><br>"We were trying every day to have every meal together. And that was very important. So for example, when somebody was running late, because we had some unscheduled, whatever activity … everybody would wait, even if we were hungry, everybody would wait for the last crew member to arrive. I think the meals were the most important part of the day, honestly."
	**Polar treks**
	"And two, were having awful turmoil, and it was pulling the team down. And one was, in my mind, pretty much bullying the other one … it was quite awful to watch … And I got really angry with the one that I thought was being unkind. So we ended up having a battle, which is fine, I don't mind conflict if I if I believe in what I've done so if I'm you know, so I found it easier to take the conflict. …And then the whole team got on. So I did start a conversation, the whole team got involved and said, Well, you know, we're all not behaving very well. So as a team, we had a conversation, we didn't address what had happened, because that had been done. We just decided to move forward and and let it go…."<br><br>"I think it's important to respect each other … And if we have a disagreement, we try to analyze the situation and understand each other's point of view. And if someone had said something that shouldn't be said, it's time to say, Okay, I'm sorry. Sorry, I said that, I didn't mean it, we understand that you were offended. And we just have to put this behind us ... start all over again, and move forward."

	"And what we did was the person at the front, would stop and wouldn't start for 10-15 minutes, whatever it was we needed on that expedition, depending on the experience. But we didn't start the time until the last person got there. Yeah. Because otherwise, the poor person at the end of the group is always catching up. And it's an awful thing to have to do … And we always pull our sledges up together. So, we were all together, we didn't stand straight … it was a full team, we made really sure."
**Team Dynamics over Time: End of the Mission**	**Antarctica**
	"I went on the ship. Because being on a plane, being probably back within 24 hours, from one of the most amazing experiences of your life. But on the streets of Cape Town, I consider it just dangerous, traffic, many people, you just can't cope with it…"<br><br>"We personalise a lot. The base. So yeah, by the end of the winter it was really our base. Yeah. And then it was a little bit frustrating to to remove everything to make it clear for, for, the newcomers."<br><br>"Because in the end, in the month of October, September, October, it's necessary to clean the base, from the roof till the till the sides, everything to prepare for the summer campaign is also one procedure for keep the base clean always. And this is, this is what is done in the end of winter."<br><br>"I mean you start off thinking it's gonna be forever. And suddenly, you've only got a couple of months left. And you think oh my Lord, I wanted to do so much of this and so much of that and then suddenly the planes arrive and then suddenly you think, oh, they're here and then you think oh, then I've got lots of time to enjoy, like the sunshine and then suddenly you're on the boat leaving and you think oh when did that happen, what happened to the last year and a half."
	**HI-SEAS**
	"Maybe a month, I don't know, and also the fear of, you know, what's going to happen to my job now because some people quit their jobs and what am I doing next and the reality of the world coming back in a way. But I tell you as soon as we left the habitat, it was everyone was ready. It was really great. Yeah, yeah. It was just it was that fear of the unknown, in my opinion, that started to worry people. And then the group wasn't quite as, as, as cohesive as it had been probably for the rest of the mission. But once once we kind of had jumped over that that hurdle. And we were in the debrief week, everyone was just like normal again."<br><br>"… it was like the worst debriefing ever … we asked that they weren't being asked a lot of technical questions, by the end by people in there was like three people had engineering backgrounds. And they

	just, they all the things that we were worried about with the data collection. They're just like, Oh, yeah, it's fine like this … it didn't seem like they understood what they were doing with the instrument. And it was just, it was bad. It was one of the worst was probably the worst debrief I've I've ever been in.”
	**MDRS/FMARS**
	“So, the idea was to do two flights in one day. So, I went out with half the staff, all the rubbish at me, and we got to resolute and the weather turned really bad and it couldn't fly back. So, the other people were sort of left at the station for another night without any communications not clear when the plane was going to come back if we had crashed or whatever. So yeah, and there I was having a nice hot shower, enjoying a meal, with all like cooked vegetables and you know, they're out there. Yes. But you know, they were happy the next day. So that was good.”<br><br>“The saddest time was yeah, saying goodbye at the end to everybody… yeah, there were tears. And everyone was quite emotional, we had hugs, and everyone was quite emotional.”
**Gender-related Issues**	“I know that be a woman in Concordia is not easy. neither in the summer neither in the winter ... Try to not to, to say I'm a women's … I'm a feminist so we have to do like this. Is not good, because you are you know, yes, you have to be you have to defend your ideas, but you have to go to compromise your ideas also with the other because the other guys are more more more men than women …You have to say your idea but you don't have to be extreme but also in the other and the other part of the men point of view of course. Yeah. So at the end, you have to compromise yourself.”<br><br>“one crew member when I first arrived, he wasn't in our winter crew, but he asked for me to be sent home because he didn't think that it was appropriate that ESA has chosen a girl to, because I was the first one to be the ESA doctor. And he also thought that I was an experiment in myself to see what would happen by putting a young girl in the crew.”<br><br>“So this is also what's what's going on now. I mean, they take it as it comes. They try to always have women there. But if it turns out that they only have one or two, they would always ask them: Are you prepared to be the only or the only of two? Yeah, and if they decline, then they get a chance next year”<br><br>“So was one situation of jealousy, something like this, and mixed with isolation and some conflicts that was already in the base. And then we never understand what really happened in his mind. And we don't understand he was trying to make a suicide or something like this.”

	"X and Y got together, got engaged, really soon. And so, the situation was clear to everyone, and it was normal. We saw them really happy, really satisfied and so it was a pleasure for all of us to see them like this."<br><br>"And I mean, you've got lots of stuff, one of the guys would ask for a blowjob every single day … But, um, so I did find it hard being a girl in that environment, I think, definitely."<br><br>"But I mean, for example, something that was said so often that it didn't even register … So this was something that was said so frequently that it didn't even register with me. And it's only now in retrospect that I'm like, Oh my god, you know, that was such an insulting thing to say."<br><br>"Yeah, this sort of sexual politics on base were a real thing. And they were a pretty major thing. And yet, on an official level, you were never allowed to talk about it, you know. So, I think the first problem was that from a management perspective, they thought gender equality meant not talking about it, you know, just pretending as if, you know, it wasn't there almost. And, yeah, that was the first thing that made it difficult, because obviously, when you mix men and women together, you there are our attractions, there's romance, there's all the rest of it. But even if you have all female crew or male crew, again, I suspect they're still the same kind of issues that crop up. It's just that, you know, you, the management preferred to assume that that was never an issue…"<br><br>"I think, for me, personally, some of the things I didn't expect that were a problem was that a lot of the guys if they had any kind of issues, it was kind of in their head that, you know, to go and talk about this, I need to go and talk to a woman, you know, that somehow they would get more sympathy or, you know, more hugs from, if they came to talk to a woman. And I became a bit exhausted, because I found that, you know, every time I was in the common space, so like, every Saturday night dinner, for example, it would end up with one of the guys who've had a few too many beers crying onto my shoulder about his girlfriend back home that had left him or his parents divorcing, or that, I don't know that there are a 100 different things. And, you know, to start off with, you really are soaking yourself in, and you're trying to offer constructive sympathy and advice, but I became emotionally exhausted, it was like, please can I just have a night where I don't have to listen to your problems."
	**HI-SEAS**
	"Um, so in our case, the relationships were extremely stable. So there was one relationship that was basically, essentially lasted for the whole mission, the other relationship. There was some some

	interruptions in between, but like … at least they were still close friends during the times when they were not … And so, having the relationships actually helped stabilise the crew … So, in our case, it was surprisingly and extremely stable and helped stabilising the crew and the group dynamics. Because I know from previous HI-SEAS missions that there was some mixing and like some ex-partner going to go out and walk with some some other person, which is like the worst thing that you can do. So, we had nothing like that …”<br><br>“There, there was some flirtation in our group, I don't think it had a huge impact. I definitely think it's something we should talk about, and it should be part of training and the discussion. I think we shy away from it. And that was one of those training things, we could have used more of just the realisation that feelings come up differently in isolation. But I think using kind of the same methods of conflict of just trying to not start rumours or talk behind each other's backs, talk to people directly. I think those are the most effective ways of kind of keeping both good flirtatious feelings and also negative conflict feelings in check.”
	**MDRS/FMARS**
	“I think everybody had quite high principles. Yeah. All of you know, what's appropriate, and what's not appropriate. So there was no inappropriate behaviour, between the men and the women in the group”<br><br>“All the men was either married or engaged. And all the female was singles so I'm not sure if it's relevant or not, but nothing happened, and everything was fine from this side of the human psyche.”<br><br>“Yes, to remember that men are men. So, I mean, we have different we by nature, we are different ... And I think I would say, Yeah, don't flirt, if you are not really sure that you really like this person, and, you know, you want to go further. So just to flirt, you know, don't do it just for that. Because in this tiny environment, and isolation, everything, all the problems, they are exaggerated…”<br><br>“I think we became like, no sex group. I mean, no gender group. So we didn't consider each other as men or women. We all had the same problems. We all were, you know, you can take shower only once a week. So we all didn’t look good. And that's fine, everyone ... Yeah … one of our crew members, every time when I wash my hair after one week … I came from shower, and he said, Oh … you’re a woman again!”
	**Lunares**

	“So the guys in the team were easier to trigger with some of the conflicts. And me and the other female, we were trying to keep the conflicts low.”
	**Polar treks**
	“not when it comes to mental aspects, but from a physical point of view, yes. I have had several times and we just tried to divide the way that some guys or women, most cases, women can be less strong physically. So we take some weight of the sledge and put that onto some of the stronger guys just to have the same speed so that we can move forward and to be equally tired at the end of the day. So that is one decision that you have to make them on the way that people just have to accept.”
**Inclusion of Disabled Persons**	**HI-SEAS**
	“…but in reality, it's just a matter of time until accidents are happening. So that's kind of the idea of the mission to have someone who has had an accident. I mean, in their case, okay, it was 10 years ago, and they are kind of used to being handicapped or not handicapped or disabled. But yeah, having someone on the team, so the person we had he’s blind, and he lost both his hands except for thumb and index on his right hand. And so, the question is kind of how can the crew deal with this kind of disability? What kind of tasks can he perform? How is this changing the group dynamics? How can he manage to get around in the habitat, those kind of kind of things? And from there, I think one of the I mean, it's definitely an additional challenge.”
