# Supplementary material for: Living on the edge: How to prepare for it?
Source: Front Neuroergon. 2022 Dec 14;3:1007774. doi: 10.3389/fnrgo.2022.1007774 (PMC10790891; doi:10.3389/fnrgo.2022.1007774)
Supplement: Supplementary file 5 [file Table_5.pdf]

Supplementary Table 5. Individual level. What it takes: how to survive an ICE-sojourn.		
**Communication for Conflict Solution**	23	**Antarctic**
		“The most important thing is that people have to speak each other and say … from the beginning … yeah, it's important to talk about problems before they turn into hate or anger against the person and to address the problem instead of the person.”<br><br>“And, also, you have some people that kind of naturally emerge, that can smoothen the relation of others and people that do not choose sides, people that are open to both parties of the conflict.”<br><br>“Every time I saw an improvement in some situation was because people were doing one big effort to to try to communicate each other”<br><br>“… they are always complaining about things, they are always creating problems instead of solving them. And they are always trying to say bad words in the back of the other people. And I think that this behaviour is totally wrong for a place like [that]”
		**HI-SEAS**
		“so one thing that helped a lot was having a crew member who just cracks jokes even at very inappropriate times. You know, nothing's better you know, like everyone is like fighting, like having a big argument. And then someone just says something totally stupid and you can't help but laugh that just releases the tension. So that is extremely helpful. I mean, it doesn't really solve problems. But it helps relieving the space for for moment and like, sometimes, like usually when, when that happened, it gave the opportunity for everyone to just, you know, mentally take a step back and think about Okay, what are we actually fighting about? What are we actually arguing about?”<br><br>“There was a study where they kind of counted the number of times people in isolation communicated. And you can start to see in the paper, the idea yeah, right. And so it was like, it was very indicative of, you know, healthy crew has a lot of one on one communication, and they don't really say causation there. But there's certainly a correlation. And so I really pushed to implement a lot of one on one communication, not just you know, once a group, but I really tried to encourage people to build those one on one relationships, and I tried my best to do that myself. Obviously, you get along with some people better than with others. And so it takes a little bit of effort with some. And it was a little bit more difficult, you know, for a couple of people, but once you just get over the awkwardness of it in the beginning, it actually pays off a lot.”

**Being Curious**	7	**Antarctica**
		"…to be interested in what the others do, and this aspect can really help, like I was telling before So, be interested in astronomy, what glaciologists do … just go help others when there is some free time."
**Mutual Investment and Helpfulness**	12	**Antarctica**
		"If you invest in helping others, then gradually those people will return the efforts. Or they may help you without you doing something first, but then it would be good if you return to cultivate the relationship …you need effort on both sides."<br><br>"But for example, if the cook is having a problem with the food, yeah, you can help him. And so you will pass that day to with with the cook, for example, preparing food."<br><br>"Try to help other people. Sometimes they might approach you anyway, it was a question, but sometimes you just need to observe all of this. You just need to develop this sense. Do they want to be approached? Do they need to be approached? Yeah. Or do you have to propose something."
		**MDRS**
		"… we were lucky because from the beginning, we all supported each other. And we understood that only us nothing else we have. So, you need to make the environment as comfortable as it can be, and trying to work with each other, and be like a family."
		**HI-SEAS**
		"One of the strengths of our mission, in my opinion, was the empathy that each of our crew members had for each other. I think that was a very strong theme … not everyone wanted the same things emotionally, which you think would cause problems, but really helped a lot with resolving them because someone always understood you. If one person didn't understand there was someone else there that might … for me, knowing each person cared a lot about my feelings and how I was doing, even if they weren't the most sociable or vocal…"
**Respecting Group Process and Put the Group above Yourself**	12	**Antarctica**
		"And it should mention it in after the week on the glacier that the questions, are you prepared to go to Antarctica with this group? Not just you. But do you agree to take everyone else? Yeah, this question there's no, if you like, there's no psychological screening that would test anyway."

		I think … always listening is the most important thing, I guess. And so you have to build that team, you have to build a team on which you are sure that those people are able to listen for the whole mission … like in a relation, couple … a relationship between two people. You can be happier, happier only if you think, at the other before of you. So, if you take care of people, people will take care of you. Yeah. So you, you come in a second place, you know, if you are able to behave like this, you you win…”
		**MDRS/FMARS**
		“One thing that was problematic was that we had one crew member who never acknowledged when, when … made a mistake. And I mean, then it becomes difficult … it's still difficult to to to argue or explain yourself to someone who doesn't even acknowledge that they might have made a mistake.”<br><br>“the thing that worked for me and for the crew is that I was honest enough to recognise my mistakes and correct them before we make it make the situation unbearable.”
**Keeping Positive**	9	**Antarctica**
		“I hope I did a lot of things with the target to improve others life and my life, because of course, seeing seeing other people happy. It's only one satisfactory way to live the mission. If you … if you can make people happy. You are doing the the good things, because you also know that you won't have problems you won't have complaints. So I tried. sometimes, I was good. Sometimes, I was I wasn't.”
		**MDRS/FMARS**
		“It may sound very superficial, but it it's really helping being positive, even if you don't feel like being positive. Because if you I mean, you're only six people, you know, you're getting up and you're in a bad mood and … then you're spreading it and then of course you're affecting each other and you're dragging each other down. And so just you know being positive even though it doesn't Yeah, even, maybe you don't really feel like it, but it helps. I mean, it even helps tune yourself up, just like when you look in the mirror and you smile at yourself, like, after a certain point, you just can't help but smile back.”
		**Lunares**
		“So I really try to observe how to make people more happy because if they are happy, they will do exactly what I need from them. So this is exactly what I've done.”
**Down to Earth, and Being Aware of**	12	**Antarctica**
		“So if you go along for the roller coaster ride, you’re a dead weight, you make it more difficult for those who are trying to put effort and to help.”

**Isolation Effects**		"Yeah, sometimes the problem is, you and not, the other one, and you need to be prepared to accept that."<br><br>"Sometimes I was trying to help to remember that all that happened there, it's especially because of isolation. So, I was trying to use the same, the same mantra that I was telling to myself, to explain to others …. I mean, because of the isolation, people start to feel bad. And not always they associate this feeling bad to the fact that they are isolated. So, they search the reason in other people, they say, Okay, if I feel bad is because of this person … they not always people are able to think, okay, it's it's one very extreme situation. We are isolated. And for this reason, I feel frustrated. (P5)<br><br>"Don't expect it to be amazing. Don't expect it to be awful. It's probably somewhere in the middle for everyone."
		**MDRS/FMARS**
		"Yeah, yeah. Well, it's like, in a family, you know, you cannot say okay, you pissed me off and I'm walking the street and I'm, I'm going to another family. You know. We were in the middle of the Arctic, and we were like the six souls in 1000 kilometres around. You have not much of a choice. Yeah."
**Accepting Yourself – It is and There is no Escape**	5	**Antarctica**
		"You might, you will learn probably a lot about yourself, but you shouldn't go there to look for yourself. If you're here and you're trying to get away from something, this is probably not a good place to be. Because at some point, you might want to go back and then you can't go back."<br><br>"So I think accepting yourself, all sides of yourself and try to get to know yourself as good as possible before you leave is a good advice."
**Professional Ethics and Attitude**	8	**Antarctica**
		"… going there so that you can really prove that you know your stuff, because there's no one there that can help you."<br><br>"So not necessarily just what we wanted to get out the mission, but we want, what we wanted, everyone to get out of the mission. And we decided to prioritise crew cohesion and good data."
		**Polar Trek**

		"Because we wanted to help scientists and we were working with scientists around the world. And that was what gave us the, if you will, the motivation to keep going when it was really bad …"<br><br>"There are people that have sponsored you, there are people out there that you've got to service, there are people relying on you. And it's okay to fail. But you can't say I gave in, you took this job, nobody asked you to do it, you took it. So, you have a duty."
**Interest in Space**	5	**MDRS/FMARS**
		"So, if you really take this as your work is, and the only thing why you're here is for your science goal, or any other goals that related to you know, space exploration. So, you take it seriously… "
